# A Robust CS-DOA Method for Multi-UAV-Assisted Agricultural Vehicle Localization in Smart Tillage

**DOI:** 10.3390/s26175377

**Published:** 2026-08-25

**Authors:** Jingyao Zhang, Ningning Ma, Heyang Li, Yancong Wang, Xiaobo Zhang, Zaiwang Lu, Lei Li, Haihua Chen, Yucheng Zhang

**Affiliations:** 1 Institute of Computing Technology, Chinese Academy of Sciences, Beijing 100190, China; zhangjingyao23p@ict.ac.cn (J.Z.); liheyang23p@ict.ac.cn (H.L.); wangyancong@ict.ac.cn (Y.W.); zhangxiaobo24b@ict.ac.cn (X.Z.); luzaiwang21b@ict.ac.cn (Z.L.); lilei22p@ict.ac.cn (L.L.); 2 University of Chinese Academy of Sciences, Beijing 100049, China; 3 Institute of Applied Ecology, Chinese Academy of Sciences, Shenyang 110164, China; ma_ningning23@163.com

**Keywords:** smart tillage, unmanned ground vehicle (UGV) localization, unmanned aerial vehicle (UAV), direction-of-arrival (DOA) estimation, compressed sensing (CS), local overcomplete dictionary (LOD), particle swarm optimization (PSO)

## Abstract

The advancement of precision agriculture and smart tillage relies on high-precision, real-time perception of unmanned ground vehicle (UGV) positions. In large-scale farmland operations, conventional Global Navigation Satellite System (GNSS)-based positioning may suffer from short-term signal loss, degrading accuracy. Multi-unmanned aerial vehicle (UAV)-assisted vehicle localization based on direction-of-arrival (DOA) estimation can provide critical positioning compensation for UGV, where compressed sensing (CS)-based DOA-assisted localization algorithms are commonly employed. However, existing schemes neither account for the bias induced by the local positional oscillation of UAVs, nor address the limited accuracy and real-time performance of CS-based DOA estimation, restricting their agricultural deployment. To this end, this paper first develops an assisted-localization architecture that explicitly incorporates the local positional offsets of multiple UAVs, together with a corresponding array signal reception model. To overcome the accuracy–efficiency trade-off of conventional CS-DOA methods, an adaptive local overcomplete dictionary (LOD) is then constructed to robustly refine the angular resolution around the region of interest. With the number of sources *K* assumed to be known and fixed, a particle swarm optimization (PSO)-based local refinement algorithm is further introduced to adaptively optimize the DOA estimates within the constructed local dictionary, thereby improving estimation robustness under low-SNR and coherent-source conditions. Consequently, the proposed method improves robustness while maintaining favorable localization accuracy and computational efficiency in the simulated scenarios. Simulation results show that it substantially reduces localization error compared with state-of-the-art algorithms, suggesting its potential as a localization-assistance approach for UGV navigation in sustainable tillage.

## 1. Introduction

Under the combined pressures of intensifying global climate change, continuous population growth, and increasingly scarce arable land, climate-smart agriculture (CSA) [1,2] has become a core paradigm for driving the sustainable transformation of agriculture. Technological innovation, represented by precision agriculture, is widely regarded as an important pathway toward achieving CSA goals. Within the technical framework of precision agriculture, the automatic navigation of unmanned ground vehicle (UGV) (e.g., agricultural machinery and spraying vehicles) is recognized as a core supporting component, playing an irreplaceable role in improving production efficiency and operation quality while reducing resource consumption. In particular, high-precision and low-latency positioning of unmanned vehicles is the key to enabling automatic navigation. At present, positioning based on the global navigation satellite system (GNSS) is the primary approach [3,4], such as GPS [5]. Conventional GNSS-assisted positioning methods typically rely on densely deployed infrastructure, such as base stations (BSs) [6] or access points (APs) [7], to achieve high-precision positioning through real-time kinematic (RTK) [8] techniques. However, in large-scale farmland scenarios, the autonomous operation of unmanned vehicles faces two practical limitations. First, BSs and APs are sparse. The sparse infrastructure prevents agricultural machinery from receiving stable RTK correction information, causing the positioning accuracy to degrade sharply—typically from the centimeter level to the meter level. Second, 4G/5G network coverage is poor. In large-scale agricultural scenarios, weak signal strength, limited backhaul links, and narrow channel bandwidth lead to significant transmission delays of positioning correction information, which can easily result in short-term loss of positioning data.

To ensure precise and real-time positioning of unmanned vehicles when the GNSS signal is briefly lost, several assisted positioning architectures have been proposed to compensate for the missing positioning information.

Multi-sensor fusion-based assisted positioning architectures can combine the advantages of different sensors for joint vehicle positioning, achieving higher accuracy and stability than standalone positioning methods [9,10,11]. However, to attain the required accuracy, these methods rely on redundant sensor installation, as each intelligent device must be equipped with multiple sensors, which substantially increases the system cost.

With the development of unmanned aerial vehicle (UAV) technology, assisted positioning architectures that employ multiple UAVs as “mobile point base stations” have been proposed as an alternative to fixed and dense ground infrastructure. Such architectures offer more flexible and convenient deployment and have been widely adopted. An assisted location analysis framework for vehicle positioning in smart-city industrial zones has been proposed [9], which consists of distributed mobile edge computing and a UAV-deployed Internet of Things to enable flexible positioning. A location-based robust beamforming algorithm has been proposed [10], which fuses UAV position and mobility information to correct direction-of-arrival (DOA) estimation errors and optimize communication and positioning performance. A source localization method for multi-UAV networks based on a deep neural network and Cramér–Rao bound (CRB) weighting has been proposed [11], which improves positioning accuracy and computational efficiency through the DeepSSF network and a weighted fusion strategy. A cooperative positioning system employing multiple UAVs equipped with monostatic multiple-input multiple-output (MIMO) radar has been proposed [12], which achieves high-precision maritime target localization in a space-air-ground integrated network (SAGIN) environment based on weighted subspace fitting. A three-dimensional reconstruction strategy based on multi-angle observations from UAV-swarm synthetic aperture radar (UAV-SAR) has been proposed [13] for tracking vehicle targets. A multi-UAV cooperative positioning architecture based on an interactive multiple-model maneuvering-target tracking algorithm has been proposed [14], which is applicable to UAV platforms with bearing-only measurements.

However, **Problem 1:** existing frameworks all assume that UAVs remain stationary during the positioning process. In practical positioning scenarios, environmental factors such as strong winds cause local positional deviations (oscillations) of the UAVs, which severely degrades the target positioning accuracy.

In addition, the design of the positioning algorithm is another important aspect of the assisted positioning architecture. In multi-UAV-assisted positioning architectures, positioning algorithms fall into four main categories. First, time-of-arrival/time-difference-of-arrival (TOA/TDOA)-based algorithms [15,16] require strict time synchronization between the UAVs and ground devices and are highly sensitive to clock offsets—a requirement that is difficult to satisfy in farmland with poor 4G/5G coverage. Second, received-signal-strength-indicator (RSSI)-based algorithms [17] suffer severe accuracy loss from multipath fading in terrain-obstructed environments, typically achieving only meter-level accuracy. Third, fingerprint-based algorithms [18] rely on extensive offline field surveys, which is impractical for vast farmland. Fourth, DOA based algorithms [19] require only a compact uniform linear array (ULA) at the receiver, do not need strict time synchronization, and can achieve sub-degree angular accuracy with only a moderate number of antenna elements. Considering the practical constraints of agricultural deployment, DOA estimation algorithms strike the best balance among accuracy, hardware cost, and deployment feasibility. Therefore, this algorithm is adopted in this paper.

Generally, DOA estimation algorithms can be divided into two categories according to their sampling frequency. The first category comprises algorithms that follow the Nyquist sampling rate. The beamforming method [19] and Capon’s method [20] serve as fundamental algorithms for DOA estimation; however, their estimation accuracy is limited and cannot meet the accuracy requirements of practical systems. To address this problem, subspace decomposition algorithms such as MUSIC [21,22,23] and ESPRIT [24] have been proposed for DOA estimation. These algorithms are widely used in practical systems because they offer acceptable estimation accuracy and low computational complexity under high SNR and sufficient snapshots. However, MUSIC and ESPRIT have poor capability in handling coherent signals and must rely on preprocessing techniques [23] to assist estimation. Therefore, the FBSS-ESPRIT algorithm [25] has been proposed, which can process incoherent signals with good real-time performance but suffers from poor estimation accuracy. To overcome this limitation, subspace fitting algorithms such as stochastic maximum likelihood (SML) [26,27] and weighted subspace fitting (WSF) [28] have been proposed. These algorithms achieve higher accuracy than subspace decomposition algorithms and can also handle coherent signals simultaneously. However, they involve a multidimensional nonlinear optimization problem, resulting in extremely high computational complexity that often prevents their direct application to practical systems.

To address this issue, various optimization algorithms based on subspace fitting have been proposed. A robust block-sparse reconstruction DOA estimation algorithm based on WSF (MUC-WSF) has been proposed [12]; by parameterizing the mutual coupling manifold matrix and constructing an optimal weighted subspace fitting model, it achieves sparse reconstruction without aperture loss under unknown mutual coupling and improves the accuracy and robustness of multi-UAV cooperative maritime target positioning in SAGIN environments. A sieve maximum likelihood method (SiML) has been proposed [29]; by extending the SML criterion to an infinite-dimensional functional data model and using a sieve to constrain the subspace dimension, it effectively overcomes the high computational cost of conventional SML and its limitation to point-source models, achieving efficient, unbiased, and asymptotically efficient estimation of arbitrarily shaped sources. A stochastic maximum likelihood estimation algorithm based on the alternating direction method of multipliers (MESA) has been proposed [30]; by reformulating the SML problem as a rank-constrained Toeplitz covariance estimation and solving it with a sequential alternating direction method of multipliers (ADMM), it enables the localization of more uncorrelated sources than sensors in sparse linear arrays and provides robust estimation for highly correlated and coherent source scenarios, while theoretically proving the robustness of this SML method to source correlation. Although these algorithms reduce the computational complexity, the problem of real-time performance still remains.

With the continuous development of DOA estimation technology, the signals received by the array carry an increasing amount of information, and signal processing requires an ever-wider bandwidth, which in turn drives the Nyquist sampling rate ever higher. Consequently, traditional DOA estimation algorithms based on the Nyquist sampling theorem impose increasingly demanding requirements in practical applications. Therefore, how to reduce the sampling frequency—or find an algorithm that operates at a rate far below the Nyquist frequency while still recovering the original information—has attracted widespread attention from researchers.

Building on the above, the second category of algorithms achieves DOA estimation by sampling the signal at a rate far below the Nyquist frequency. Algorithms that exploit the sparse representation of signals are known as compressed sensing (CS) algorithms [31,32,33,34,35]. In practical farmland positioning scenarios, the limited cost budget for signal-reception hardware restricts the number of signal samples (i.e., snapshots), resulting in insufficient information about the original signal. Therefore, this paper adopts the CS algorithm as the assisted positioning algorithm. The CS algorithm involves three key aspects: sparse representation, compressive sampling, and signal reconstruction.

At present, according to the completeness of the dictionary, the sparse representation stage can be classified into three types: on-grid [31], off-grid [32], and gridless [33]. The off-grid and gridless methods avoid grid constraints, but they introduce additional floating-point operations into the overall DOA estimation system, leading to excessively high computational complexity and poor positioning timeliness. Therefore, this paper focuses on the on-grid case. On-grid algorithms sparsify the signal by constructing a sparse basis matrix from an uniform overcomplete dictionary (UOD). An algorithm based on iterative dictionary learning has been proposed, which alleviates the grid-mismatch problem in DOA estimation by alternately updating the dictionary and performing sparse recovery [31]. A two-stage fast matching pursuit (TSFMP) algorithm has been proposed [34] to reduce computational complexity and improve the positioning accuracy of multi-target DOA estimation. A generalized spatio-temporal coprime sampling (GSTCS) method has been proposed [35], aiming to maximize the degrees of freedom and jointly estimate the DOA and Doppler frequency through compressed sensing.

**Problem 2:** The method of constructing the sparse basis matrix from a UOD performs sparsification over the entire angular domain. Because the angles of incident waves are random, DOA estimation must traverse the entire angular domain. Consequently, higher positioning accuracy requires a larger dictionary size, which adversely affects the real-time performance of the positioning system.

Compressive sampling stage: The signal is first sparsified in the sparse representation stage, and the sparsified signal is then passed through the measurement matrix in the compressive sampling stage to obtain the signal measurements. To guarantee the uniqueness of the reconstruction result, the measurement matrix and the sparse basis matrix must be mutually incoherent, that is, they must satisfy the restricted isometry property (RIP) [36,37].

In the signal reconstruction stage, the orthogonal matching pursuit (OMP) algorithm [38] is commonly used for signal recovery. However, the OMP algorithm suffers from low angular resolution and an inability to distinguish highly correlated grids, resulting in poor DOA estimation accuracy. To overcome these limitations, the focused OMP (FOMP) [39] and generalized OMP (GOMP) [40] algorithms have been developed, which partially address the DOA estimation accuracy problem. In addition, the MSCM-OMP algorithm, based on the signal covariance matrix and multiple sub-covariance matrices, has been proposed [41]; it significantly improves angular estimation accuracy at low SNR while reducing computational complexity. For real-time super-resolution direction-of-arrival estimation in automotive radar sensors, the maximum likelihood real-time super-resolution (MARS) algorithm has been proposed [42]; by exploiting the data correlation between adjacent time slots to shrink the search space and employing the OMP algorithm in place of exhaustive search to efficiently solve the maximum likelihood objective function, it achieves millisecond-level real-time and high-accuracy DOA estimation.

**Problem 3:** The OMP algorithm and its variants encounter a source-correlation problem when resolving multiple signal sources. Specifically, the reconstruction accuracy of each signal source depends on the reconstruction result of the preceding source. If a reconstruction error occurs, the entire DOA estimation system may fall into a local optimum.

To address the above problems, this paper makes four main contributions:

1. A DOA-estimation-based multi-UAV-assisted positioning architecture is proposed for large-scale smart-farming cultivation scenarios. For the first time, the architecture takes UAV oscillation into account, deriving an assisted-positioning error model together with the associated positioning constraints, thereby providing systematic theoretical support for practical positioning deployment.
2. A displacement-aware array signal reception model that integrates the spatio-temporal information of UAVs is constructed. In this paper, the UAV position and transmission instant are encapsulated into the data payload to eliminate the transmit–receive geometric mismatch, and the instantaneous position is decomposed into a nominal hovering component and a Gaussian random displacement component. For the first time, within a unified framework, we explicitly reveal the mechanism by which UAV jitter affects positioning accuracy through two coupled paths—the delay domain and the angular domain—thereby establishing an analytically tractable physical modeling foundation for the subsequent DOA estimation algorithm.
3. An adaptive local overcomplete dictionary (LOD) model is proposed to improve the real-time performance of positioning. This paper adopts a two-stage “coarse-estimation–local-refinement” dictionary construction strategy: the closed-form solution of FBSS-ESPRIT is first used as the prior center, after which a fine grid is generated only within its neighborhood. Innovatively taking the RMSE as the basic grid unit, we reveal—through Monte Carlo experiments—the phase-transition behavior of the dictionary-width coefficient as a function of SNR, and propose a constrained, SNR-adaptive piecewise-constant value criterion. Based on this criterion, the adaptive LOD model is constructed.
4. A multi-source decoupled signal reconstruction algorithm based on particle swarm optimization (PSO) is proposed. Departing from the traditional serial matching-pursuit paradigm, this paper reformulates the joint DOA estimation of multiple sources as a swarm-intelligence optimization problem in which the angle combination of all sources serves as a single particle state vector. This method transforms the originally serially coupled coherent-source estimation into parallel and independent optimization at the particle level, and, by leveraging the continuous search capability of the PSO algorithm within the narrow LOD domain, breaks through the grid-quantization limitation to achieve high-accuracy DOA estimation. The final results demonstrate that the proposed joint optimization algorithm offers lower computational complexity and higher DOA estimation accuracy, and that within the assisted positioning architecture it achieves a lower positioning error than existing algorithms.

The remainder of this paper is organized as follows. The proposed multi-UAV-assisted positioning architecture and positioning error model are introduced in Section 2. The proposed signal reception model based on the local position offset of UAVs is presented in Section 3. The proposed compressed-sensing DOA estimation algorithm optimized by the LOD-PSO model is described in Section 4. The simulation results are provided in Section 5. The experimental results are discussed in Section 6. The conclusions are drawn in Section 7.

## 2. System Architecture

**Figure 1:**
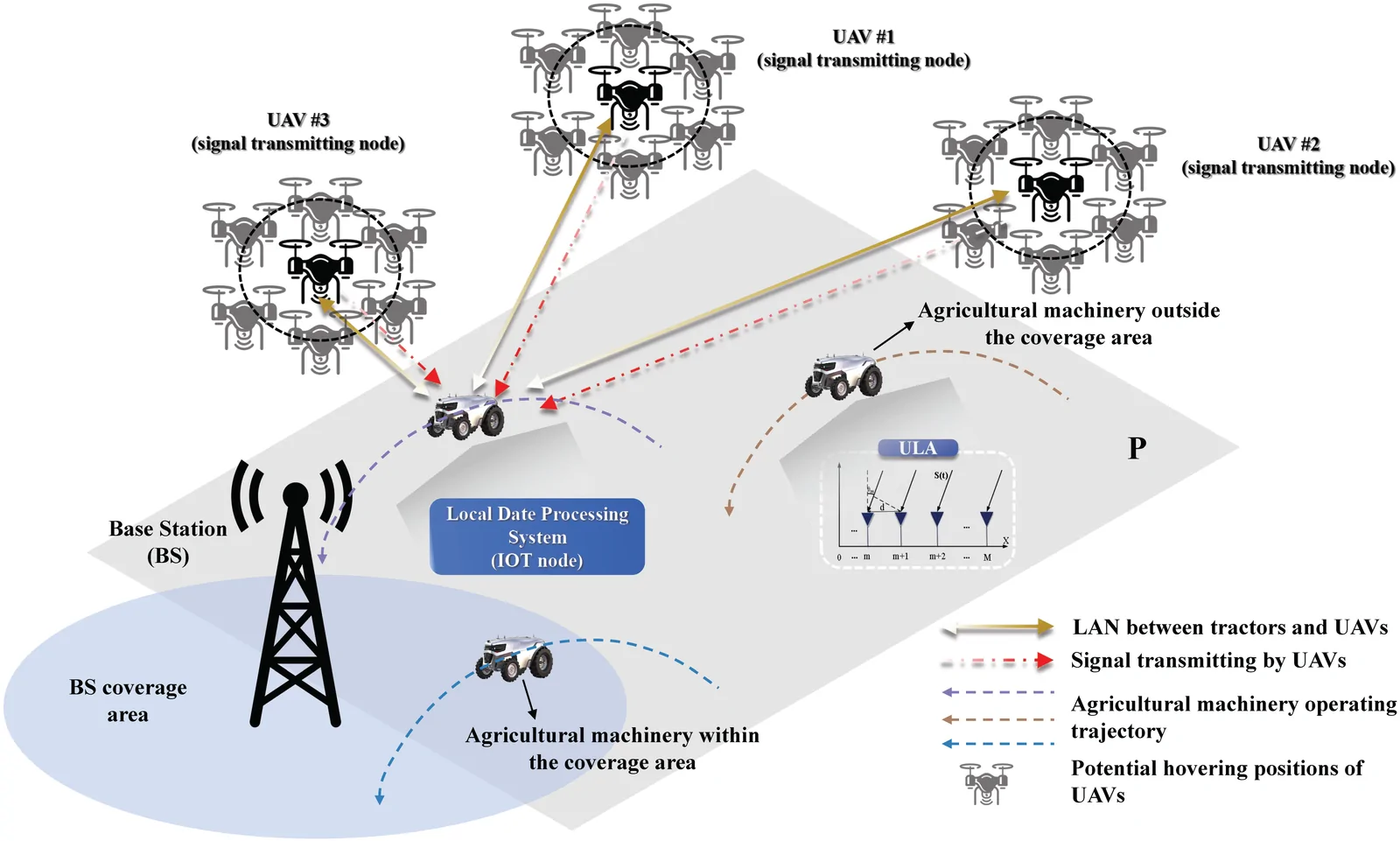
A multi-UAV-assisted positioning architecture based on DOA estimation for farmland localization scenarios.

Based on the capability of UAVs to assist target positioning and navigation in SAGIN scenarios, a multi-UAV-assisted positioning architecture based on DOA estimation is constructed for farmland positioning scenarios in this paper, as illustrated in Figure 1. P denotes the working area of the agricultural equipment, and the position of the agricultural equipment V is located within P . Each UAV serves as a signal transmission node, with positions denoted as $U_{1},\;U_{2}$ , and $U_{3}$ , respectively. Each piece of agricultural equipment follows an independent working trajectory and is equipped with a ULA for signal reception [43,44]. It is worth noting that the ULA only needs to operate in the sub-6 GHz band, and the half-wavelength inter-element spacing results in an aperture of only a few centimeters. Each array element can be implemented using a standard patch antenna. In addition, the receiving chain of the ULA requires only a conventional Software Defined Radio (SDR) level RF front end, including a low-noise amplifier, mixer, filter, and ADC, without multi-sensor integration or any additional precision components on the ground equipment. Therefore, the unit hardware cost of the ULA is significantly lower than that of a survey-grade RTK-GNSS receiver or a light detection and ranging–inertial measurement unit (LiDAR–IMU) fusion module.

The UGV and unmanned aerial vehicles (UAVs) communicate via a local area network. The yellow lines represent information exchange between the UAVs and the UGV, including the real-time UAV position, battery level, and timestamp. The UAVs only need to transmit radio signals, as indicated by the red lines. The UGV is equipped with a local data-processing system and can process the signals transmitted by the UAVs locally, without forwarding them to the mobile edge computing (MEC) server. This avoids localization-information transmission latency caused by poor network quality and limited signal bandwidth. It should be noted that no signal interference exists between the two communication links of the UGV and UAVs because the communication bands are isolated in frequency.

In Figure 1, the blue region denotes the coverage area of the base station. When the UGV enters the base-station coverage area, the RTK-GNSS positioning method is adopted to perform conventional unmanned operations. When the UGV moves outside the base-station coverage area, the accuracy of conventional positioning methods decreases substantially. Therefore, a multi-UAV-based DOA estimation-assisted localization algorithm is adopted for cooperative localization. When the assisted localization algorithm is used to localize the UGV, each UAV fuses multi-sensor data, including RTK-GNSS and IMU data [45], and the resulting localization information can typically be output at a frequency of 50Hz [46]. However, after the electromagnetic signal is transmitted to the agricultural machinery and processed by the local data-processing system, the localization frequency of the agricultural machinery is typically lower than 10Hz .

The accuracy target adopted here stems from concrete tillage and seeding operations. Row following in seeding and inter-row mechanical weeding typically demand a lateral accuracy of 2– 10cm , whereas broadcast spraying, fertilization, and coarse tillage can tolerate 10– 50cm . During brief RTK-GNSS outages, the assisted localization layer proposed in this paper is intended not to replace RTK but to constrain drift, keeping the implement within the agronomic tolerance of the operation and thereby preventing the skips and overlaps that waste seed, chemicals, and fuel. Since a UGV traveling at a typical operating speed of 1– 3m/s moves 0.1 – 0.3m per 0.1s frame, the sub-meter errors reported in Section 5.4, delivered at the equipment rate of below 10Hz , are sufficient to keep medium-to-coarse operations within tolerance during such outages.

**Figure 2:**
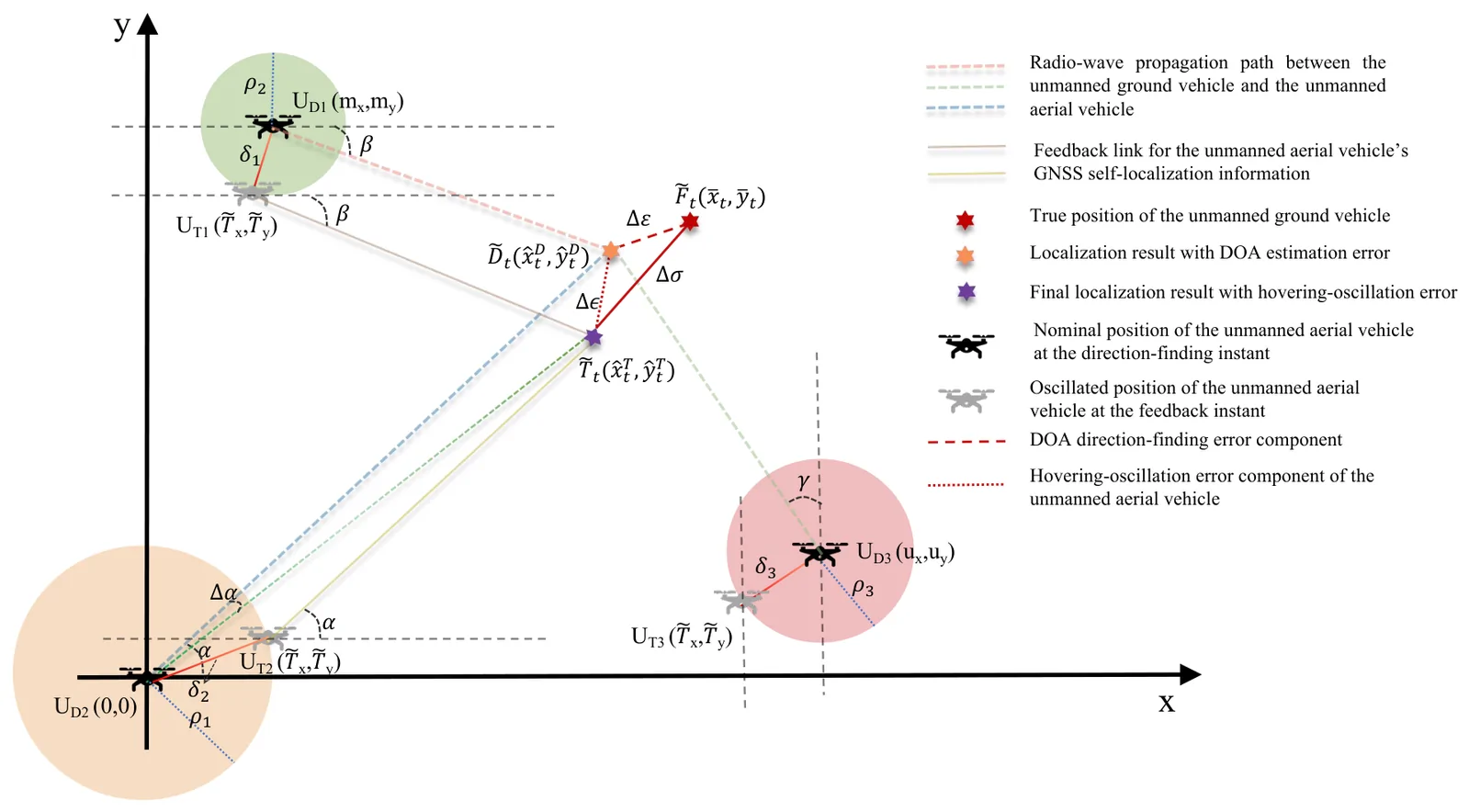
Schematic diagram of the localization architecture.

In practical localization scenarios, environmental factors such as strong winds may cause local position deviations of the UAVs. After a UAV transmits an electromagnetic signal to the agricultural machinery, the agricultural machinery processes the received signal and calculates the DOA estimate. Owing to the frequency mismatch between the UAV transmission rate and the signal-processing rate of the agricultural machinery, when the DOA value is calculated and the RTK-GNSS position of the UAV is required to derive the coordinates of the agricultural machinery, the UAV position may already have shifted. Consequently, the transmitted RTK-GNSS position no longer corresponds to the original position from which the electromagnetic signal was transmitted, resulting in localization errors. The specific architectural schematic is shown in Figure 2.

In a multi-UAV cooperative direction-finding localization system, each platform can obtain only the DOA of the target, so the two-dimensional position of the target must be determined by the spatial intersection of several lines of sight (LOS). The localization accuracy, however, depends not only on the direction-finding algorithm itself but also on the relative geometric configuration of the UAVs, the angle-measurement errors, and the hovering jitter of the platforms. In this work we first establish the linear intersection model derived from the azimuth observations together with the least-squares position estimate; we then develop, on this basis, a first-order propagation model that maps the angle-measurement error and platform jitter to the final localization error; and finally we summarize the geometric feasibility constraints that the relative positions of the three UAVs must satisfy.

To facilitate the subsequent derivations, the principal notation is defined as follows. The position of the *k*-th UAV is denoted $U_{Dk}=\left(U_{kx},\;U_{ky}\right)$ with k=1, 2, 3 ; without loss of generality UAV 2 is taken as the coordinate origin, i.e., $U_{D2}=\left(0,\;0\right)$ , and the coordinates of UAV 1 and UAV 3 are written $U_{D1}=\left(m_{x},\;m_{y}\right)$ and $U_{D3}=\left(u_{x},\;u_{y}\right)$ , respectively. The true position of the target is $p={\left({\bar{x}}_{t},\;{\bar{y}}_{t}\right)}^{T}$ , and its least-squares estimate is $\hat{p}$ . The azimuth measured by the *k*-th UAV is $\varphi_{k}$ , its noisy observation is ${\hat{\varphi }}_{k}$ , and the range from the *k*-th UAV to the target is $R_{k}=∥p-U_{Dk}∥$ .

Each UAV $U_{Dk}$ obtains the azimuth $\varphi_{k}$ of the target through direction finding. In the two-dimensional plane, this azimuth defines a line of sight (a ray) originating at $U_{Dk}$ and pointing toward the target; any point (x, y) on the LOS satisfies the direction-consistency condition

(1) $$\frac{y-U_{ky}}{x-U_{kx}}=\tan\varphi_{k},\;$$

where (x, y) are the coordinates of a running point on the LOS, $U_{kx}$ and $U_{ky}$ are the abscissa and ordinate of the *k*-th UAV, and $\varphi_{k}$ is its measured azimuth. Equation (1) contains the tangent function and becomes singular as $\varphi_{k}\to \pi /2$ . To remove this numerical singularity and obtain a linear form valid for all azimuths, we cross-multiply and rearrange it into

(2) $$\left(x-U_{kx}\right)\sin\varphi_{k}-\left(y-U_{ky}\right)\cos\varphi_{k}=0,\;$$

with symbols as in (1). Expanding and moving the constant term to the right-hand side yields the standard linear equation in the target coordinates,

(3) $$x\sin\varphi_{k}-y\cos\varphi_{k}=U_{kx}\sin\varphi_{k}-U_{ky}\cos\varphi_{k},\;$$

in which the left-hand side is a linear combination of the target coordinates and the right-hand side is a constant determined solely by the position and azimuth of the *k*-th UAV.

To simplify the exposition, define the unit normal vector of the *k*-th LOS,

(4) $$n_{k}={\left(\sin\varphi_{k},\;\;-\cos\varphi_{k}\right)}^{T},\;$$

where $n_{k}$ is perpendicular to the direction of the *k*-th LOS and satisfies $∥n_{k}∥=1$ . With this definition, (3) can be written compactly as

(5) $$n_{k}^{T}p=n_{k}^{T}U_{Dk}≜b_{k},\;$$

where $p={\left({\bar{x}}_{t},\;{\bar{y}}_{t}\right)}^{T}$ is the true target position and $b_{k}=n_{k}^{T}U_{Dk}$ is the constant term of the *k*-th LOS equation, determined jointly by the UAV position and its azimuth. Stacking the LOS equations (5) of the three UAVs gives an over-determined linear system

(6) $$Ap=b,\;\;A=\left[\begin{array}{cc} \sin\varphi_{1} & -\cos\varphi_{1}\\ \sin\varphi_{2} & -\cos\varphi_{2}\\ \sin\varphi_{3} & -\cos\varphi_{3} \end{array}\right],\;\;b=\left[\begin{array}{c} b_{1}\\ b_{2}\\ b_{3} \end{array}\right],\;$$

where $A\in R^{3\times 2}$ is the observation matrix, each row of which is the LOS normal $n_{k}^{T}$ , and $b\in R^{3}$ is the constant vector with components $b_{k}$ as defined in (5).

In the noise-free case (6) holds exactly and the three lines of sight are concurrent; in practice, however, the azimuths are perturbed to ${\hat{\varphi }}_{k}$ , the three LOS no longer intersect at a single point, and a least-squares criterion must be adopted. Defining the residual vector r=Ap−b and minimizing its squared Euclidean norm ${∥r∥}^{2}$ , the normal equations yield the least-squares estimate of the target position,

(7) $$\hat{p}={\left(A^{T}A\right)}^{-1}A^{T}b,\;$$

where $\hat{p}$ is the estimated target position and $A^{T}A\in R^{2\times 2}$ is the information matrix. This estimate exists and is unique if and only if A has full column rank, i.e., the information matrix is nonsingular, whose geometric meaning is that the three UAVs are not collinear. To quantify the influence of the geometric configuration on localization accuracy, and under the simplifying assumption that the LOS observation errors are independent and identically distributed, we define the geometric dilution of precision (GDOP),

(8) $$\mathrm{GDOP}=\sqrt{\mathrm{tr}\;\left[{\left(A^{T}A\right)}^{-1}\right]}=\sqrt{\frac{3}{\sum_{i<j}\sin^{2}\left(\varphi_{i}-\varphi_{j}\right)}},\;$$

where tr[·] denotes the matrix trace and $\varphi_{i}-\varphi_{j}$ is the direction difference between the *i*-th and *j*-th lines of sight. A smaller GDOP indicates a better geometric configuration; as the three UAVs tend to collinearity, $\sum_{i<j}\sin^{2}\left(\varphi_{i}-\varphi_{j}\right)\to 0$ , the GDOP diverges, and the localization error is amplified without bound. The least-squares estimate (7) and the geometric-accuracy measure (8) together form the basis for the error-propagation analysis that follows.

Building on the observation model and the least-squares solution, we next analyze how two classes of error sources—angle-measurement error and platform jitter—propagate to the final localization error. Collecting the azimuth errors of the three UAVs into the vector $\Delta \varphi ={\left[\Delta \varphi_{1},\;\Delta \varphi_{2},\;\Delta \varphi_{3}\right]}^{T}$ , where $\Delta \varphi_{k}={\hat{\varphi }}_{k}-\varphi_{k}$ is the angle-measurement error of the *k*-th UAV. Consider first the case in which the UAV positions retain their nominal values while only the azimuths are perturbed. Taking the first-order total differential of the LOS constraint function $f_{k}\left(p\right)=n_{k}^{T}\left(p-U_{Dk}\right)$ (whose form derives from (5)) and enforcing that it remains identically zero, we obtain

(9) $$n_{k}^{T}\;\vec{\Delta \varepsilon }+R_{k}\;\Delta \varphi_{k}=0,\;$$

where $\vec{\Delta \varepsilon }$ is the localization offset induced by the angle-measurement error and $R_{k}=∥p-U_{Dk}∥$ is the range from the *k*-th UAV to the target. The coefficient $R_{k}$ arises from $\partial f_{k}/\partial \varphi_{k}$ : differentiating the normal vector $n_{k}$ with respect to the azimuth gives exactly the LOS direction unit vector $u_{k}={\left(\cos\varphi_{k},\;\sin\varphi_{k}\right)}^{T}$ , which, combined with the geometric relation $p-U_{Dk}=R_{k}u_{k}$ , yields the result. Stacking the three equations (9) into the matrix form $A\;\vec{\Delta \varepsilon }=-D\;\Delta \varphi$ and taking the least-squares solution gives

(10) $$\vec{\Delta \varepsilon }=-{\left(A^{T}A\right)}^{-1}A^{T}D\;\Delta \varphi =J_{\varphi }\;\Delta \varphi ,\;$$

where $D=\mathrm{diag}(R_{1},\;R_{2},\;R_{3})$ is the diagonal matrix of the three ranges and $J_{\varphi }=-{\left(A^{T}A\right)}^{-1}A^{T}D\in R^{2\times 3}$ is the angular Jacobian matrix. The corresponding expected mean-square localization error is

(11) $$E[∥\vec{\Delta \varepsilon }{∥}^{2}]=\mathrm{tr}\;\left(J_{\varphi }C_{\varphi }J_{\varphi }^{T}\right)=\mathrm{tr}\;\left[{\left(A^{T}A\right)}^{-1}A^{T}DC_{\varphi }DA{\left(A^{T}A\right)}^{-1}\right],\;$$

where $C_{\varphi }=\mathrm{diag}\left(\sigma_{\varphi_{1}}^{2},\;\sigma_{\varphi_{2}}^{2},\;\sigma_{\varphi_{3}}^{2}\right)$ is the angle-error covariance matrix and $\sigma_{\varphi_{k}}^{2}$ is the angle-measurement variance of the *k*-th UAV. The *k*-th diagonal element of the inner factor $DC_{\varphi }D$ equals $R_{k}^{2}\sigma_{\varphi_{k}}^{2}$ , showing that the localization error is proportional to the product of the squared range and the angle-measurement variance, while the outer factor ${\left(A^{T}A\right)}^{-1}$ is governed by the GDOP of (8). This term depends only on the direction-finding accuracy and the geometric configuration, and is independent of time.

Within the total latency Δt from direction finding to reporting, each UAV undergoes a displacement $\delta_{k}=U_{Tk}-U_{Dk}$ due to hovering jitter, where $U_{Tk}$ is the actual UAV position after the latency. From the partial derivative of the constraint function with respect to the UAV position, $\partial f_{k}/\partial U_{Dk}=-n_{k}^{T}$ , an analogous stacking yields the localization offset due to jitter,

(12) $$\vec{\Delta \epsilon }={\left(A^{T}A\right)}^{-1}A^{T}w_{\mathrm{osc}},\;\;w_{\mathrm{osc},k}=n_{k}^{T}\delta_{k},\;$$

where $\vec{\Delta \epsilon }$ is the localization offset induced by jitter and $w_{\mathrm{osc}}$ is the vector of jitter projections onto the LOS normals. This shows that only the jitter component perpendicular to the LOS injects localization error, acting equivalently as an angular perturbation of $\delta_{k}^{⊥}/R_{k}$ . Assuming the jitter is isotropic and mutually independent across UAVs, with normal-projection variance $\sigma_{\mathrm{osc},\;k}^{2}\Delta t^{2}$ , the expected mean-square localization error is

(13) $$E[∥\vec{\Delta \epsilon }{∥}^{2}]=\mathrm{tr}\;\left[{\left(A^{T}A\right)}^{-1}A^{T}\mathrm{diag}\left(\sigma_{\mathrm{osc},\;k}^{2}\right)A{\left(A^{T}A\right)}^{-1}\right]\Delta t^{2}\propto \sum_{k=1}^{3}\frac{\sigma_{\mathrm{osc},\;k}^{2}}{R_{k}^{2}}\Delta t^{2},\;$$

where $\sigma_{\mathrm{osc},k}^{2}$ is the velocity variance of the *k*-th UAV’s jitter and Δt is the total latency. This term is proportional to the square of the latency, indicating that any processing and communication delay is amplified by the platform jitter into an irreducible localization error, which is precisely why the system imposes a hard real-time constraint. The detailed derivation is provided in Appendix A.

The final localization error is the vector sum of the two terms above, $\vec{\Delta \sigma }=\vec{\Delta \varepsilon }+\vec{\Delta \epsilon }$ . Since the angle-measurement noise and the platform jitter are statistically independent, the expected cross-covariance term vanishes, and the expected total mean-square error decouples into two independent components,

(14) $$E[∥\vec{\Delta \sigma }{∥}^{2}]=\underset{\mathrm{accuracy}\;\mathrm{term}}{\underbrace{\sum_{k=1}^{3}\frac{\mathrm{CRLB}(\varphi_{k})}{\eta_{k}}{∥\frac{\partial \hat{p}}{\partial \varphi_{k}}∥}^{2}}}+\underset{\mathrm{real}-\mathrm{time}\;\mathrm{term}}{\underbrace{\sum_{k=1}^{3}\frac{\sigma_{\mathrm{osc},\;k}^{2}}{R_{k}^{2}}\Delta t^{2}}},\;$$

where $\mathrm{CRLB}(\varphi_{k})$ is the Cramér–Rao lower bound of the *k*-th UAV’s angle measurement, $\eta_{k}\in \left(0,\;1\right]$ is the efficiency factor of the direction-finding algorithm, and $\partial \hat{p}/\partial \varphi_{k}$ is the *k*-th column of the Jacobian $J_{\varphi }$ . Equation (14) decomposes the localization error rigorously into two indispensable sources: the accuracy term is determined jointly by the direction-finding efficiency and the geometric configuration (the GDOP), and it approaches its theoretical lower limit when $\eta_{k}\to 1$ and the deployment is optimal; the real-time term is proportional to the square of the total latency, so that even with perfectly accurate angle measurement, any delay still injects error through (12). The two constitute an intrinsic trade-off—a more complex algorithm may raise the efficiency $\eta_{k}$ but increases the processing latency Δt —and therefore an optimal cooperative-localization algorithm must jointly minimize both.

The error expressions (11) and (14) remain bounded only under the premise that the three-UAV configuration is well conditioned. Accordingly, we summarize the geometric feasibility constraints that the relative positions of the three UAVs must satisfy; together, these constraints delimit the feasible deployment region over which the least-squares localization of (7) is solvable, stable, and of controllable accuracy. Setting aside the physical-feasibility constraints related to detection and communication, the purely geometric constraints can be summarized in three interrelated conclusions.

**Constraints**:

1. The three UAVs must not lie on the same straight line, i.e., the area of the triangle they span must be strictly positive,
(C1) $$S_{▵}=\frac{1}{2}\left|m_{x}u_{y}-m_{y}u_{x}\right|>0,\;$$
  where $S_{▵}$ is the area of the triangle formed by the three UAVs, $m_{x},\;m_{y}$ are the coordinates of UAV 1 relative to the origin (taken at UAV 2), and $u_{x},\;u_{y}$ are those of UAV 3.
2. The observation matrix A must have full column rank, and the condition number of its information matrix must not exceed a prescribed threshold,
(C2) $$\det\left(A^{T}A\right)=\sum_{i<j}\sin^{2}\left(\varphi_{i}-\varphi_{j}\right)\neq 0,\;\;\kappa \left(A^{T}A\right)=\frac{\lambda_{\max}}{\lambda_{\min}}\leq \kappa_{\max},\;$$
  where $\lambda_{\max}$ and $\lambda_{\min}$ are the largest and smallest eigenvalues of the information matrix and $\kappa_{\max}$ is the maximum admissible condition number.
3. The intersection angle of any two UAV lines of sight must avoid the ill-conditioned region near 0 or π and fall within a safe interval,
(C3) $$\theta_{\min}\leq \left|\varphi_{i}-\varphi_{j}\right|\leq \pi -\theta_{\min},\;\;\forall \;i\neq j,\;\;\theta_{\min}=\arcsin\left(1/G_{\max}\right),\;$$
  where $\theta_{\min}$ is the minimum admissible intersection angle and $G_{\max}$ is the maximum admissible geometric amplification factor.

The threshold $\kappa_{\max}$ introduced in (C2) is the key parameter determining the size of the feasible region. Regarding its value, the choice of $\kappa_{\max}$ is essentially a compromise between localization accuracy and deployment flexibility, and in practice it is usually determined by back-solving from the tolerable error-amplification factor $G_{\max}=\sqrt{\kappa_{\max}}$ . Common values fall roughly into three tiers. In high-accuracy scenarios with stringent requirements, $\kappa_{\max}$ is often taken between 4 and 10, corresponding to an intersection angle no smaller than about $30^{\circ }$ – $40^{\circ }$ and a geometric amplification of about 2–3 times, for which the error ellipse is fairly close to a circle. In general engineering scenarios it may be relaxed to $\kappa_{\max}\approx 10$ –100, corresponding to an intersection angle no smaller than about $10^{\circ }$ – $20^{\circ }$ , in exchange for deployment flexibility. In practical systems, $\kappa_{\max}=10$ is often adopted as a robust yet not overly conservative default threshold, with the final value still to be calibrated in light of the angle-measurement accuracy $\sigma_{\varphi_{k}}$ , the target ranges $R_{k}$ (cf. (11)), and the mission’s accuracy specification.

## 3. Signal Reception Model Based on UAV State Variations

Assume that the number of array elements is *M*. In theory, the array manifold can be arbitrary. However, in practical DOA estimation, the array model is typically configured as a specific array geometry, such as a ULA. Furthermore, it is assumed that *K* far-field narrowband signals impinge on the sensor array from distinct directions $\theta_{1},\;\theta_{2},\;\ldots ,\;\theta_{K}$ relative to the reference point, and the center frequencies of these signals are known. The signals are modeled as far-field narrowband signals, and M>K .

The baseband complex signal transmitted by the *k*-th UAV at time instant $t_{t}^{\left(k\right)}$ is expressed as follows:

(15) $$s_{k}\left(t\right)=c_{k}\left(t-t_{t}^{\left(k\right)}\right)\cdot e^{j\theta_{d,\;k}\left(t-t_{t}^{\left(k\right)}\right)},\;\;t\in \left[t_{t}^{\left(k\right)},\;t_{t}^{\left(k\right)}+T_{\mathrm{frame}}\right].$$

where $s_{k}\left(t\right)$ denotes the baseband complex signal transmitted by the *k*-th UAV. $c_{k}\left(\cdot \right)$ represents the known pilot/ranging sequence, which is used for signal synchronization, time delay estimation, and DOA estimation. $t_{t}^{\left(k\right)}$ represents the transmission time instant of the *k*-th signal. $T_{\mathrm{frame}}$ denotes the duration of the signal frame. $\theta_{d,\;k}\left(\cdot \right)$ denotes the data modulation phase, which carries the encoded data packet. The $D_{k}$ is defined as follows:

(16) $$D_{k}\left(t_{t}^{\left(k\right)}\right)=\left\{{\mathrm{ID}}_{k},\;x_{U_{k}}\left(t_{t}^{\left(k\right)}\right),\;y_{U_{k}}\left(t_{t}^{\left(k\right)}\right),\;z_{U_{k}}\left(t_{t}^{\left(k\right)}\right),\;t_{t}^{\left(k\right)},\;\mathrm{CRC}\right\},\;$$

where ${\mathrm{ID}}_{k}$ denotes the unique identifier of the *k*-th UAV. $x_{U_{k}}\left(t_{t}^{\left(k\right)}\right),\;y_{U_{k}}\left(t_{t}^{\left(k\right)}\right),\;z_{U_{k}}\left(t_{t}^{\left(k\right)}\right)$ represent the three-dimensional position of the UAV at the transmission time instant $t_{t}^{\left(k\right)}$ . CRC denotes the Cyclic Redundancy Check code, which is employed to ensure data integrity.

The signal arrives at the agricultural equipment array after propagating through the wireless channel. The steering vector for the signal direction is given by the following:

(17) $$a\left(\theta_{k}\right)={\left[1,\;e^{-j\frac{2\pi d}{\lambda }\cos\theta_{k}},\;\ldots ,\;e^{-j\frac{2\pi (M-1)d}{\lambda }\cos\theta_{k}}\right]}^{T}.$$

where $a\left(\theta_{k}\right)\in C^{M\times 1}$ is the steering vector corresponding to the direction $\theta_{k}$ . $\theta_{k}=\varphi_{k}-\psi$ denotes the relative azimuth angle of the *k*-th signal with respect to the heading direction of the agricultural equipment. $\varphi_{k}$ denotes the absolute azimuth angle of the *k*-th signal in the global coordinate system. ψ denotes the yaw angle (heading angle) of the agricultural equipment. *d* is the inter-element spacing of the array, which is typically set to half a wavelength, i.e., d=λ/2 . λ denotes the carrier wavelength of the signal.

At the reception time instant $t_{r}$ , the baseband signal received by the m-th array element is the superposition of all *k*-th signals and noise, which is expressed as follows:

(18) $$\begin{array}{cc} x_{m}\left(t_{r}\right) & =\sum_{k=1}^{K}\alpha_{k}\cdot s_{k}\left(\Delta {\hat{t}}_{k}\right)\cdot e^{-j\frac{2\pi }{\lambda }\left(m-1\right)d\cos\theta_{k}\left(t_{t}^{\left(k\right)}\right)}+n_{m}\left(t_{r}\right),\;\\ \Delta {\hat{t}}_{k} & =t_{r}-t_{t}^{\left(k\right)}-\tau_{k},\;\\ \tau_{k} & =\frac{∥p_{U_{k}}\left(t_{t}^{\left(k\right)}\right)-p_{T}\left(t_{t}^{\left(k\right)}\right)∥}{c}. \end{array}$$

where $x_{m}\left(t_{r}\right)$ denotes the baseband complex signal received by the *m*-th array element at time instant $t_{r}$ . $\alpha_{k}$ denotes the complex amplitude of the *k*-th signal, incorporating the path loss and the random channel phase. $\Delta {\hat{t}}_{k}$ denotes the effective time argument of the received baseband signal after compensating for the transmission timestamp and the propagation delay. The variable $t_{r}$ denotes the signal reception time at the agricultural equipment array, $t_{t}^{\left(k\right)}$ denotes the transmission time of the signal emitted by the *k*-th UAV, and $\tau_{k}$ denotes the propagation delay from the *k*-th UAV to the receiving array. $p_{U_{k}}\left(t_{t}^{\left(k\right)}\right)$ denotes the position of the UAV at the transmission time instant. $p_{T}\left(t_{t}^{\left(k\right)}\right)$ denotes the position of the agricultural equipment at the transmission time instant, which is retrospectively obtained from the historical positioning data of the agricultural equipment. Since the propagation delay is much smaller than the motion timescale of the agricultural equipment, $p_{T}\left(t_{t}^{\left(k\right)}\right)$ is used as an approximation of the receiver position during signal propagation. *c* denotes the speed of light. $n_{m}\left(t_{r}\right)$ denotes the additive noise at the *m*-th array element, which is typically modeled as complex white Gaussian noise.

To make the physical mechanism of UAV oscillation mathematically explicit in Equation (18), the time-varying UAV position $p_{U_{k}}\left(t_{t}^{\left(k\right)}\right)$ is further decomposed into a nominal hovering component and a stochastic local displacement component:

(19) $$p_{U_{k}}\;\left(t_{t}^{\left(k\right)}\right)={\bar{p}}_{U_{k}}+\Delta p_{U_{k}}\;\left(t_{t}^{\left(k\right)}\right),\;$$

where ${\bar{p}}_{U_{k}}={\left[{\bar{x}}_{U_{k}},\;\;{\bar{y}}_{U_{k}},\;\;{\bar{z}}_{U_{k}}\right]}^{T}$ denotes the nominal hovering position reported by the UAV’s onboard GPS/IMU fusion module at 50Hz , and $\Delta p_{U_{k}}\left(t_{t}^{\left(k\right)}\right)$ represents the wind/vibration-induced local positional displacement of the UAV. The displacement is statistically modeled as a zero-mean Gaussian process

(20) $$\Delta p_{U_{k}}\;\left(t_{t}^{\left(k\right)}\right)∼N\;\left(0,\;\;\sigma_{p}^{2}\;I_{3}\right),\;$$

where the displacement intensity $\sigma_{p}$ characterizes the severity of environmental disturbance. In farmland scenarios, $\sigma_{p}$ typically ranges from a few centimeters under calm conditions to tens of centimeters under strong-wind conditions. The case $\sigma_{p}=0$ reduces to the conventional stationary-UAV assumption adopted by existing works, whereas $\sigma_{p}>0$ corresponds to the practical scenario considered in this paper.

Substituting Equation (19) into the propagation delay defined in Equation (18), the displacement-aware delay is obtained as follows:

(21) $$\tau_{k}=\frac{∥\;{\bar{p}}_{U_{k}}+\Delta p_{U_{k}}\;\left(t_{t}^{\left(k\right)}\right)-p_{T}\;\left(t_{t}^{\left(k\right)}\right)\;∥}{c}.$$

Equation (21) is mathematically consistent with the original definition of $\tau_{k}$ in Equation (18); it merely makes the previously implicit displacement component explicit. Because the UAV displacement also perturbs the geometric direction from the UAV to the agricultural equipment, the relative azimuth $\theta_{k}$ acquires a small perturbation $\Delta \theta_{k}$ . A first-order Taylor expansion gives the following:

(22) $$\begin{array}{cc} {\tilde{\theta }}_{k} & \approx \theta_{k}+\Delta \theta_{k},\;\\ \Delta \theta_{k} & ={\left(\nabla_{p_{U_{k}}}\;\theta_{k}\right)}^{T}\Delta p_{U_{k}}\;\left(t_{t}^{\left(k\right)}\right),\; \end{array}$$

so that the perturbed steering vector can be expressed as follows:

(23) $$\begin{array}{c} a\;\left({\tilde{\theta }}_{k}\right)=a\left(\theta_{k}\right)+\frac{\partial a(\theta_{k})}{\partial \theta_{k}}\;\Delta \theta_{k}+O\;\left(\Delta \theta_{k}^{2}\right). \end{array}$$

The $\nabla_{p_{U_{k}}}\theta_{k}$ represents the gradient of $\theta_{k}$ with respect to the UAV position vector $p_{U_{k}}$ , indicating how the angle $\theta_{k}$ changes with small variations in the UAV position. The $O(\Delta \theta_{k}^{2})$ represents the higher-order terms whose magnitude is on the order of $\Delta \theta_{k}^{2}$ or smaller, which are usually neglected under small-angle perturbation assumptions. Equations (19)–(23) constitute the displacement-aware refinement of the reception model. They explicitly reveal two coupled error pathways through which UAV oscillation degrades positioning accuracy: a delay-domain error introduced via Equation (21), and an angular-domain error introduced via Equations (22) and (23). Both pathways are absent from the conventional stationary-UAV formulation, which directly motivates the displacement-aware reception model proposed in this paper.

By stacking the signals from all *M* array elements into a vector, the compact array received signal model is obtained as follows:

(24a) $$x\left(t_{r}\right)=\sum_{k=1}^{K}\alpha_{k}a\;\left(\theta_{k}\left(t_{t}^{\left(k\right)}\right)\right)s_{k}\;\left(\eta_{k}\left(t_{r}\right)\right)+n\left(t_{r}\right)$$

(24b) $$x\left(t_{r}\right)=A\;\left(\Phi (t_{t})\right)\mathrm{diag}\left(\alpha \right)s\;\left(\eta (t_{r})\right)+n\left(t_{r}\right)$$

(24c) $$\eta \left(t_{r}\right)=t_{r}1_{K}-t_{t}-\tau$$

(24d) $$\eta_{k}\left(t_{r}\right)=t_{r}-t_{t}^{\left(k\right)}-\tau_{k}$$

(24e) $$s\;\left(\eta (t_{r})\right)≜{\left[s_{1}\;\left(\eta_{1}\left(t_{r}\right)\right),\;s_{2}\;\left(\eta_{2}\left(t_{r}\right)\right),\;\ldots ,\;s_{K}\;\left(\eta_{K}\left(t_{r}\right)\right)\right]}^{T}$$

(24f) $$A\;\left(\Phi (t_{t})\right)=\left[a\;\left(\theta_{1}\left(t_{t}^{\left(1\right)}\right)\right),\;a\;\left(\theta_{2}\left(t_{t}^{\left(2\right)}\right)\right),\;\ldots ,\;a\;\left(\theta_{K}\left(t_{t}^{\left(K\right)}\right)\right)\right]$$

(24g) $$\Phi \left(t_{t}\right)={\left[\theta_{1}\left(t_{t}^{\left(1\right)}\right),\;\theta_{2}\left(t_{t}^{\left(2\right)}\right),\;\ldots ,\;\theta_{K}\left(t_{t}^{\left(K\right)}\right)\right]}^{T}$$

(24h) $$t_{t}={\left[t_{t}^{\left(1\right)},\;t_{t}^{\left(2\right)},\;\ldots ,\;t_{t}^{\left(K\right)}\right]}^{T}$$

(24i) $$\tau ={\left[\tau_{1},\;\tau_{2},\;\ldots ,\;\tau_{K}\right]}^{T}$$

where $\eta (t_{r})$ denotes the effective time-argument vector of the received UAV signals after compensating for the transmission timestamps and propagation delays. Specifically, the *k*-th element of $s(\eta \left(t_{r}\right))$ is given by $s_{k}\left(\eta_{k}\left(t_{r}\right)\right)=s_{k}\left(t_{r}-t_{t}^{\left(k\right)}-\tau_{k}\right)$ . This notation indicates that different UAV signals generally have different transmission timestamps and propagation delays. $A\;\left(\Phi (t_{t})\right)$ denotes the array manifold matrix. $\Phi (t_{t})$ represents the relative azimuth angle vector. diag(α) denotes the diagonal matrix with $\alpha ={\left[\alpha_{1},\;\alpha_{2},\;\ldots ,\;\alpha_{K}\right]}^{T}$ as its diagonal entries. τ represents the propagation delay vector.

According to the continuous-time array received signal model in Equation (24b), let $f_{s}$ denote the sampling frequency and $T_{s}=1/f_{s}$ denote the sampling period. To avoid spectral aliasing, the sampling frequency should satisfy the Nyquist criterion, i.e.,

(25) $$f_{s}\geq 2B,\;$$

where *B* is the bandwidth of the received signal. Let $t_{q}=qT_{s}$ , q=1, 2, …, Q , denote the *q*-th sampling instant. Then, the discrete-time array received signal can be expressed as

(26) $$x\left[q\right]=A\left(\Phi (t_{t})\right)\mathrm{diag}\left(\alpha \right)s\;\left(\eta (t_{q})\right)+n\left[q\right].$$

By stacking *Q* snapshots column-wise, the array received data matrix is obtained as

(27) $$X=\left[x\left[1\right],\;x\left[2\right],\;\ldots ,\;x\left[Q\right]\right]=A\left(\Phi (t_{t})\right)\mathrm{diag}\left(\alpha \right)S+N,\;$$

where S denotes the source signal snapshot matrix incorporating the transmission timestamps and propagation delays, and N denotes the array noise snapshot matrix.

Based on the discrete snapshots, the theoretical covariance matrix of the array received signal is defined as

(28) $$R_{x}=E\left[x\left[q\right]x^{H}\left[q\right]\right],\;$$

where ${\left(\cdot \right)}^{H}$ denotes the Hermitian transpose. Substituting the discrete received signal model into the above expression yields

(29) $$R_{x}=A\left(\Phi (t_{t})\right)\mathrm{diag}\left(\alpha \right)R_{s,\eta }\mathrm{diag}{\left(\alpha \right)}^{H}A^{H}\left(\Phi (t_{t})\right)+R_{n},\;$$

where

(30) $$R_{s,\eta }=E\left[s\;\left(\eta [q]\right)s^{H}\;\left(\eta [q]\right)\right]$$

is the covariance matrix of the element-wise delayed source signal vector, and $R_{n}=E\left[n\left[q\right]n^{H}\left[q\right]\right]$ is the noise covariance matrix. If the noise is modeled as zero-mean additive white Gaussian noise, then $R_{n}=\sigma_{n}^{2}I_{M}$ . In practical implementation, the theoretical covariance matrix is usually approximated by the sample covariance matrix as

(31) $${\hat{R}}_{x}=\frac{1}{Q}XX^{H}=\frac{1}{Q}\sum_{q=1}^{Q}x\left[q\right]x^{H}\left[q\right],\;$$

where

(32) $$X=\left[x[1],\;x[2],\;\ldots ,\;x[Q]\right]\in C^{M\times Q}.$$

The proposed array received signal model explicitly incorporates the transmission timestamps and propagation delays, thereby reducing the model mismatch caused by processing latency in conventional methods. Moreover, the model can be incorporated into a variety of DOA estimation algorithms.

Furthermore, in practical farmland positioning scenarios, the CS algorithm is an effective approach for DOA estimation under the condition of limited snapshots. The improved CS algorithm model is presented in detail in the next chapter.

## 4. Compressive Sensing Algorithm Optimized by the LOD-PSO Model

### 4.1. Sparse Representation

**Figure 3:**
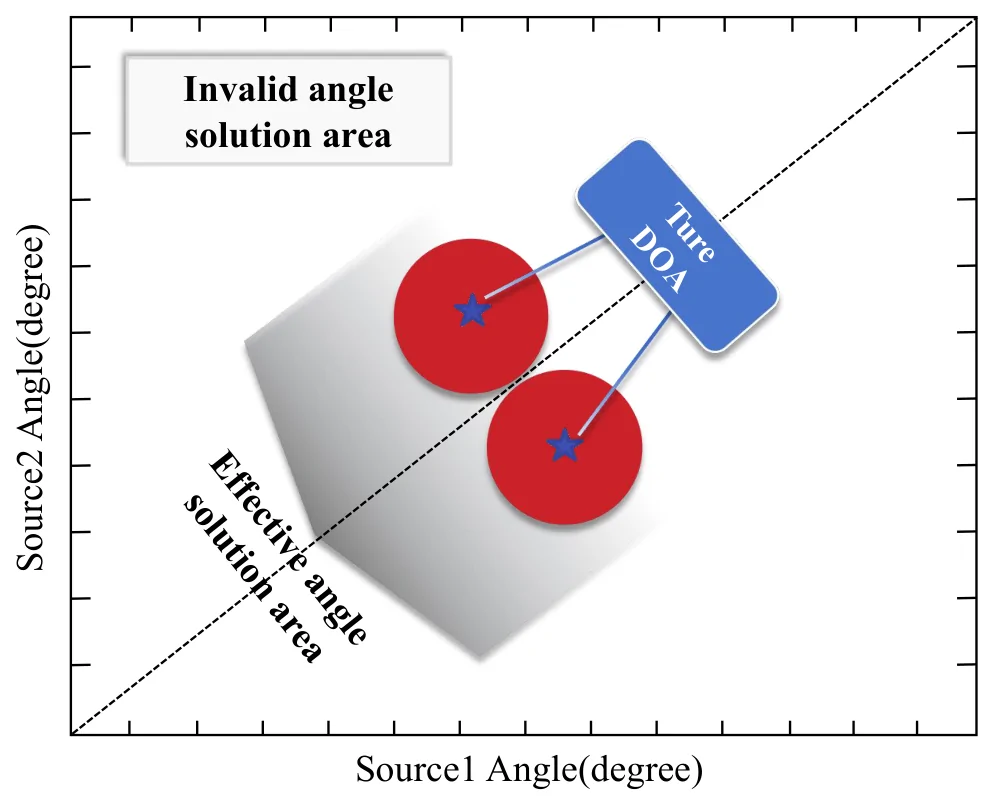
Solution-space model.

Conventional sparse representation methods typically construct a uniform overcomplete dictionary to sparsify the entire solution space. When high DOA estimation accuracy is required, the dictionary grid must be finely divided (e.g., an angular resolution of $0.01^{\circ }$ results in 18,000 grid points). Under the condition of a sparse number of sources, the grid in most angular intervals of no interest remains excessively fine, leading to a large amount of redundant computation and increasing the computational complexity of the positioning system. It is observed that the true DOA estimates can be obtained by only considering the angular interval in the vicinity of the true solution. As shown in Figure 3, the red region represents the vicinity of the true solution, referred to as the effective computation region. The remaining part corresponds to the ineffective solution space. Therefore, the key idea is reformulated as follows: whether a method can be devised to first rapidly identify the region near the true solution and then construct an overcomplete dictionary within this region to reduce computational complexity. The forward-backward spatial smoothing estimation of signal parameters via rotational invariance techniques (FBSS-ESPRIT) algorithm is characterized by fast iteration and the ability to handle both coherent and incoherent signals simultaneously. Therefore, the FBSS-ESPRIT algorithm is first employed to obtain an initial DOA estimate, which serves as prior knowledge for the construction of the overcomplete dictionary. Subsequently, a fine grid partition is performed locally in the “vicinity” of the obtained initial solution, yielding a local overcomplete dictionary (LOD) model that guarantees the inclusion of the true solution. The next step is to determine the boundary condition of the LOD model grid, i.e., to define the extent of the “vicinity.”

The DOA estimation accuracy of the FBSS-ESPRIT algorithm varies significantly with the variation of the signal-to-noise ratio (SNR), i.e., the root-mean-square error (RMSE) changes accordingly. Based on this characteristic, this paper adopts the RMSE of the angular error under different SNR conditions as the fundamental unit for grid partitioning. The RMSE of the FBSS-ESPRIT algorithm is expressed as follows:

(33) $$RMSE=\frac{1}{K}\sum_{k=1}^{K}\sqrt{\frac{1}{J}\sum_{j=1}^{J}{\left({\tilde{\theta }}_{kj}-\theta_{k}\right)}^{2}},\;$$

where *K* is the number of sources. *J* denotes the number of Monte Carlo trials, which is set to 100. $\theta_{k}$ denotes the true DOA value of the *k*-th source. ${\tilde{\theta }}_{kj}$ represents the closed-form solution of the FBSS-ESPRIT algorithm for the *k*-th source in the j-th trial.

Depending on the number of sources, the boundary range for each closed-form solution of the FBSS-ESPRIT algorithm is defined as follows:

(34) $$\begin{array}{c} {\bar{\theta }}_{1}\in \left[{\tilde{\theta }}_{1}-N\times RMSE,{\tilde{\theta }}_{1}+N\times RMSE\right],\;\\ {\bar{\theta }}_{2}\in \left[{\tilde{\theta }}_{2}-N\times RMSE,{\tilde{\theta }}_{2}+N\times RMSE\right],\;\\ \ldots \\ {\bar{\theta }}_{K}\in \left[{\tilde{\theta }}_{K}-N\times RMSE,{\tilde{\theta }}_{K}+N\times RMSE\right]. \end{array}$$

**Figure 4:**
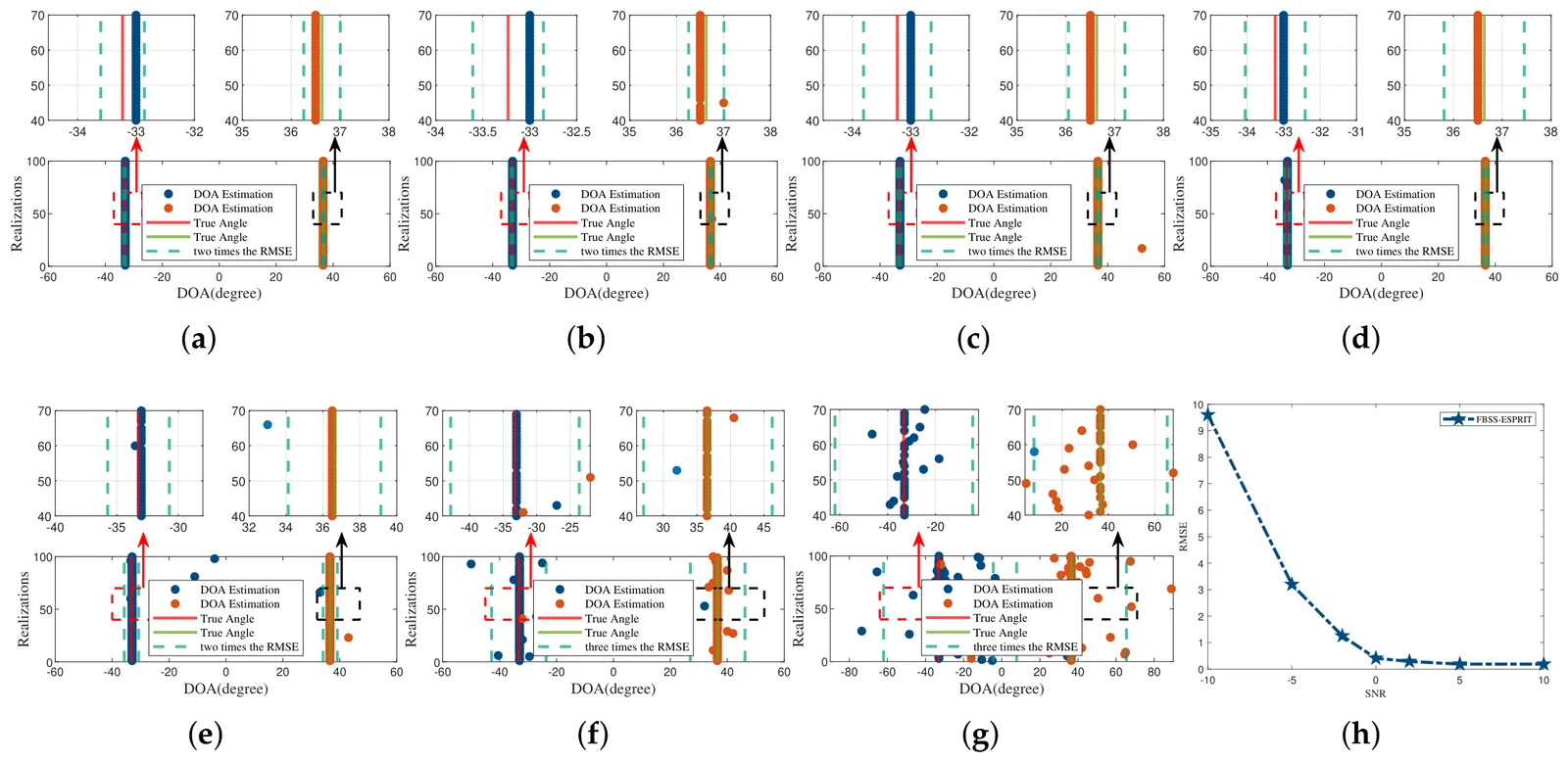
Comparison of local overcomplete dictionary boundary conditions under different SNR levels: (**a**) SNR=10 dB. (**b**) SNR=5 dB. (**c**) SNR=2 dB. (**d**) SNR=0 dB. (**e**) SNR=−2 dB. (**f**) SNR=−5 dB. (**g**) SNR=−10 dB. (**h**) RMSE vs. SNR.

As the SNR decreases, the stability of the DOA estimation by the FBSS-ESPRIT algorithm deteriorates, and the estimated DOA angle θ cannot be entirely contained within the range $\left[{\tilde{\theta }}_{k}-N\times \mathrm{RMSE},\;\;{\tilde{\theta }}_{k}+N\times \mathrm{RMSE}\right]$ . Therefore, *N* times the RMSE is required to constrain the angular space, and the selection of *N* is of critical importance. As illustrated in Figure 4, this paper presents the relationship between the DOA estimation angles of the FBSS-ESPRIT algorithm and the RMSE under SNR values of 10, 5, 2, 0, −2, −5 , and −10dB , respectively, with the number of Monte Carlo (MC) trials set to 100. The experimental conditions are set as follows: K=2 , Q=5 , and M=15 . Figure 4h shows the experimental results of the RMSE versus SNR for the FBSS-ESPRIT algorithm.

It can be observed from the figures that, when SNR≥−2dB , all estimated DOA angles successfully fall within the range of 2 times the RMSE, while when −2dB>SNR≥−10dB , all estimated DOA angles successfully fall within the range of 3 times the RMSE (in this paper, success is defined as more than 95% of the estimates falling within the interval over *J* trials). Beyond this visual observation, three systemic properties of Figure 4 directly govern the design of the LOD model.

(i) The discrete jump from N=2 to N=3 is not a smooth degradation but reflects a phase transition in subspace stability. In the regime SNR≥−2dB (Figure 4a–e), estimates remain tightly clustered around the true angles, indicating that the signal and noise eigenvectors of the FBSS-ESPRIT covariance decomposition are reliably separable. In the regime SNR<−2dB (Figure 4f,g), occasional outlier realizations appear far from the true angle, which is the signature of subspace swap events. Such outliers cannot be eliminated by additional snapshot averaging because they correspond to a qualitatively different failure mode of the eigendecomposition; the boundary condition must therefore widen discontinuously. This justifies adopting an SNR-conditioned, piecewise-constant rule for *N* rather than a single global constant.
(ii) The SNR-adaptive choice of *N* directly controls the computational budget of the downstream CS solver. The number of grid points inside the LOD scales linearly with *N*; switching from N=2 to N=3 enlarges the dictionary by 50% per source and by ${\left(1.5\right)}^{K}$ for *K* coherent sources after the Cartesian product is taken. At SNR=10dB , the LOD contains on the order of $2N\times \mathrm{RMSE}/\hat{\Delta }$ grid points per source—typically a few tens of points at an angular resolution of $0.01^{\circ }$ —versus 18, 000 points for the conventional UOD, a reduction of more than two orders of magnitude. This reduction is automatic and SNR-aware: high-SNR regimes, in which positioning is most often required, benefit from the tightest dictionary, whereas low-SNR regimes incur only a modest 50% overhead to retain robustness. From a system viewpoint, this provides a principled way to amortize computation across the SNR distribution that an agricultural UAV link actually experiences during operation.
(iii) The 95% success criterion has a direct interpretation as a bound on the per-frame positioning outage probability. Defining success as more than 95% of the realizations falling inside the LOD is equivalent to bounding the outage probability of the LOD-PSO refinement at 5% ; within the remaining frames, the true DOA lies outside the search window and cannot be recovered regardless of refinement accuracy. Because the agricultural-equipment positioning frequency is below 10Hz and the equipment moves slowly compared with the UAV update rate, such isolated misses are absorbed by the temporal smoothing of the navigation filter and do not propagate into the world-coordinate solution. This explains why the chosen *N* remains adequate for the reported simulation analysis even at SNR=−10dB , and it provides a quantitative link between the empirical curves of Figure 4 and the application-layer positioning performance.

**Figure 5:**
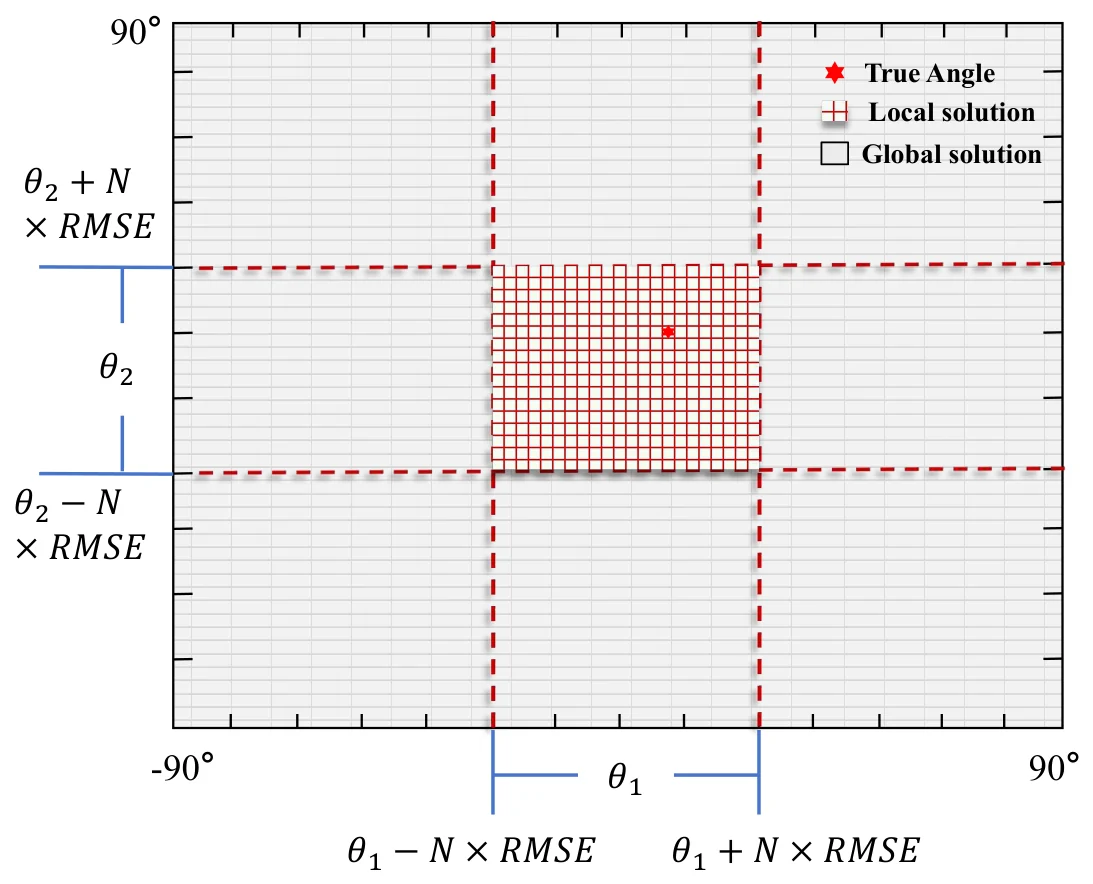
Local overcomplete dictionary model.

Based on the above analysis, the LOD is constructed, as illustrated in Figure 5.

In the figure, the red region represents the constructed LOD, within which the true solution is contained, while the gray region corresponds to the redundant computation portion eliminated by the local construction. The systemic implications of this geometry, which motivate its adoption over a conventional UOD, are summarized as follows.

The LOD changes the computational scaling of multi-source CS-DOA from exponential in the global angular domain to exponential only in a small local window. A conventional UOD with grid step $\hat{\Delta }$ over $\left[-90^{\circ },\;90^{\circ }\right]$ yields

(35) $$G_{\mathrm{UOD}}=\frac{180}{\hat{\Delta }}$$

atoms per source, and for *K* coherent sources the joint search space inflates to $G_{\mathrm{UOD}}^{K}$ . The LOD replaces $180/\hat{\Delta }$ by

(36) $$G_{\mathrm{LOD}}=\frac{2N\times \mathrm{RMSE}}{\hat{\Delta }},\;$$

which is on the order of 10 to $10^{2}$ over the SNR range of interest. The multi-source dictionary therefore shrinks by a factor of

(37) $${\left[\frac{180}{2N\times \mathrm{RMSE}}\right]}^{K},\;$$

which exceeds $10^{2K}$ at high SNR. This structural reduction is the reason the proposed pipeline can operate at the <10Hz positioning rate required by farm equipment on embedded ARM-class processors, without recourse to GPU acceleration.

Equally important, the LOD geometry decouples the coarse and fine stages of the estimator, which is the key to robustness under UAV oscillation. The coarse stage (FBSS-ESPRIT) provides an approximately unbiased center ${\tilde{\theta }}_{k}$ within whose neighborhood the true angle lies with controlled probability, but its on-grid variance alone is insufficient to meet the target accuracy. The fine stage (PSO over the LOD) refines accuracy through continuous-domain search, but only if the search window contains the truth. Figure 5 visualizes this coarse-to-fine division of labor: the red sub-rectangles constitute the working set of the fine stage, while the gray complement is provably irrelevant under the SNR-conditioned guarantee established in Figure 4. This separation is what enables the proposed displacement-aware reception model in Equation (24b) to absorb UAV oscillation without inflating the search complexity, since the position offset $p_{U_{k}}\left(t_{t}^{\left(k\right)}\right)-p_{T}\left(t_{t}^{\left(k\right)}\right)$ only perturbs the center ${\tilde{\theta }}_{k}$ , not the size of the LOD.

Finally, the LOD is a soft-fail rather than a hard-fail structure. If the coarse estimate happens to be biased by more than N×RMSE , the true solution falls outside the LOD and cannot be recovered by the fine stage. However, such events occur with probability below 5% by design, the FBSS-ESPRIT bias is approximately zero-mean across realizations, and the navigation-layer filter on the agricultural equipment can temporally smooth the DOA stream. Therefore, besides reducing computational complexity, the LOD can help confine the influence of occasional coarse-stage outliers in the simulated localization pipeline, which supports more stable localization-assistance performance in farmland-operation scenarios.

### 4.2. Signal Reconstruction

After constructing the LOD, the original CS-DOA reconstruction problem is transformed from a global sparse recovery problem over the entire angular domain into a constrained nonlinear optimization problem within a small local angular region. Therefore, the objective of the signal reconstruction stage is no longer to sequentially select dictionary atoms from the large-scale UOD, but to jointly search for the optimal multi-source angle vector within the LOD.

**Figure 6:**
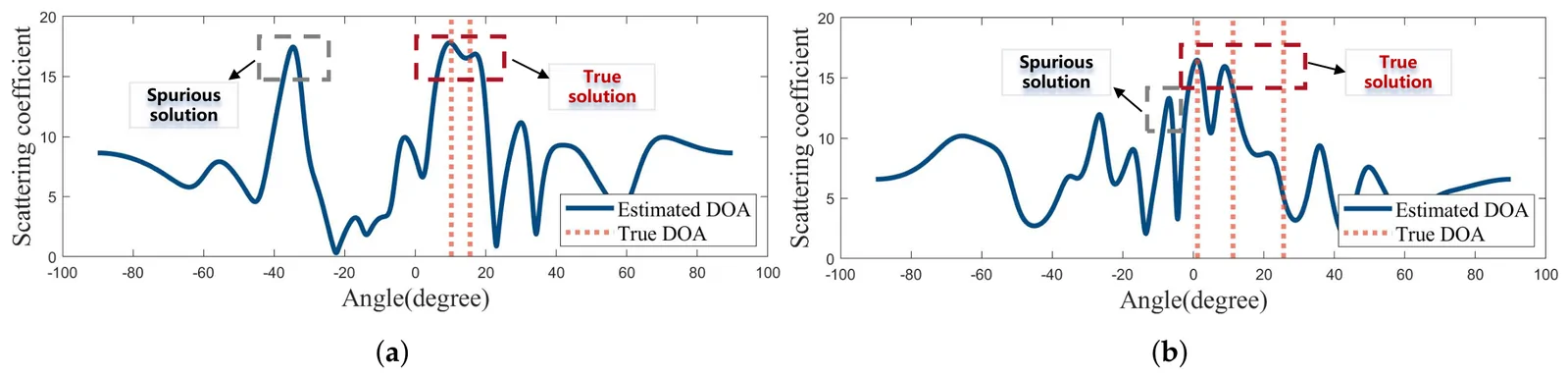
DOA estimation results based on the OMP algorithm under randomly selected incident angles: (**a**) DOA estimation result for two sources with true DOAs of $\left[10.31^{\circ },\;15.62^{\circ }\right]$ , where K=2 . (**b**) DOA estimation result for three sources with true DOAs of $\left[1.21^{\circ },\;11.29^{\circ },\;25.62^{\circ }\right]$ , where K=3 .

In the conventional OMP algorithm and its variants, the reconstruction process is inherently sequential. At each iteration, the algorithm selects the dictionary atom with the highest correlation to the current residual, subtracts its contribution, and then proceeds to estimate the next source. When two or more incident signals are highly correlated, the correlation values between dictionary atoms and the residual become coupled. Consequently, if an incorrect atom is selected in an early iteration, the direction of the current residual is altered, which directly affects subsequent atom selection. This is known as the source correlation problem. As illustrated in Figure 6, the red boxes indicate the true solutions, while the gray boxes indicate the spurious solutions. However, according to the optimization criterion of OMP, the spurious solutions are mistakenly selected as the optimal solutions. Due to the source correlation problem, the entire DOA-based positioning system fails.

The PSO algorithm possesses strong global search capability and low per-iteration computational complexity, and ot can transform the correlated source problem into an independent source problem. To address the above issue, this paper breaks through the conventional OMP framework and proposes a PSO-based signal reconstruction method, which converts the correlated source estimation into independent source estimation. In the proposed method, each particle in the swarm represents a complete candidate DOA vector rather than a single angular atom. For the *l*-th particle at the (k+1) -th iteration, its position is defined as

(38) $${\hat{\theta }}_{l}^{\prime k+1}=\left({\hat{\theta }}_{l1}^{\prime k+1},\;{\hat{\theta }}_{l2}^{\prime k+1},\;\ldots ,\;{\hat{\theta }}_{lK}^{\prime k+1}\right),\;$$

where ${\hat{\theta }}_{l}^{\prime k+1}$ denotes the candidate DOA vector represented by the *l*-th particle at the (k+1) -th iteration, $l=1,\;2,\;\ldots ,\;\tilde{P}$ is the particle index, $\tilde{P}$ is the total number of particles, *k* is the iteration index, *K* is the number of incident sources, and ${\hat{\theta }}_{li}^{\prime k+1}$ , i=1, 2, …, K , denotes the candidate DOA of the *i*-th source contained in the *l*-th particle.

The corresponding steering matrix generated by this particle is expressed as

(39) $${\hat{A}}_{l}^{\prime k+1}=\left({\hat{a}}_{l1}^{\prime k+1},\;{\hat{a}}_{l2}^{\prime k+1},\;\ldots ,\;{\hat{a}}_{lK}^{\prime k+1}\right),\;$$

where ${\hat{A}}_{l}^{\prime k+1}\in C^{M\times K}$ is the steering matrix associated with the *l*-th particle at the (k+1) -th iteration, *M* denotes the number of array elements, and ${\hat{a}}_{li}^{\prime k+1}\in C^{M\times 1}$ is the steering vector corresponding to the candidate angle ${\hat{\theta }}_{li}^{\prime k+1}$ of the *i*-th source.

Unlike the OMP algorithm, which determines the sources sequentially, the proposed method evaluates the entire *K*-source candidate angle vector simultaneously. Therefore, the reconstruction accuracy of one source no longer explicitly depends on the previously selected source. In this way, the multi-source DOA estimation problem is reformulated as a joint optimization problem, effectively suppressing the source-correlation cascade effect caused by sequential residual updating.

For each particle, the received signal is first projected onto the signal subspace generated by the corresponding steering matrix, and the complex source amplitude vector is estimated using the least-squares method. The reconstructed source coefficient vector is obtained as

(40) $$X_{l}^{\prime }={\left[{\left({\hat{A}}_{l}^{\prime k+1}\right)}^{H}{\hat{A}}_{l}^{\prime k+1}\right]}^{-1}{\left({\hat{A}}_{l}^{\prime k+1}\right)}^{H}y,\;$$

where $X_{l}^{\prime }\in C^{K\times 1}$ denotes the estimated source coefficient vector for the *l*-th particle, $y\in C^{M\times 1}$ is the received array snapshot vector.

Then, the residual between the received signal and the reconstructed signal is used as the fitness function in the PSO process, i.e.,

(41) $$\begin{array}{c} R_{l}^{\prime }=y-{\hat{A}}_{l}^{\prime k+1}X_{l}^{\prime },\;\\ fitness=∥R_{l}^{\prime }{∥}_{2},\; \end{array}$$

where $R_{l}^{\prime }\in C^{M\times 1}$ represents the reconstruction residual vector of the *l*-th particle, and fitness denotes the fitness value used to evaluate the quality of the particle.

A smaller fitness indicates that the steering matrix generated by the particle can more accurately explain the received array signal. Therefore, the PSO iteration process essentially searches within the LOD for the angle vector that minimizes the reconstruction residual.

After evaluating the fitness value of each particle, the velocity and position of the particles are updated. Specifically, for the *l*-th particle at the *k*-th iteration, the velocity vector and position vector are updated as

(42) $$v_{l}^{k+1}=\omega v_{l}^{k}+c_{1}r_{1}\left(p_{l}^{k}-{\hat{\theta }}_{l}^{\prime k}\right)+c_{2}r_{2}\left(g^{k}-{\hat{\theta }}_{l}^{\prime k}\right),\;$$

(43) $${\hat{\theta }}_{l}^{\prime k+1}={\hat{\theta }}_{l}^{\prime k}+v_{l}^{k+1},\;$$

where $v_{l}^{k}\in R^{K\times 1}$ denotes the velocity vector of the *l*-th particle at the *k*-th iteration, and $v_{l}^{k+1}$ is the updated velocity vector. The vector ${\hat{\theta }}_{l}^{\prime k}\in R^{K\times 1}$ denotes the position of the *l*-th particle at the *k*-th iteration, namely, the candidate DOA vector represented by this particle, while ${\hat{\theta }}_{l}^{\prime k+1}$ denotes its updated position. The parameter ω is the inertia weight, which controls the influence of the previous velocity on the current search direction. In this paper, the cognitive learning factor and the social learning factor are set as $c_{1}=2$ and $c_{2}=2$ , respectively. The random coefficients $r_{1}$ and $r_{2}$ are independently generated random numbers uniformly distributed in the interval [0, 1] , i.e., $r_{1},\;r_{2}∼U\left(0,\;1\right)$ . The vector $p_{l}^{k}\in R^{K\times 1}$ denotes the personal best position found by the *l*-th particle up to the *k*-th iteration, and $g^{k}\in R^{K\times 1}$ denotes the global best position found by the whole swarm up to the *k*-th iteration.

In this study, since each particle represents a complete *K*-source DOA vector, both the velocity vector and the position vector are *K*-dimensional. The *i*-th element of $v_{l}^{k}$ controls the update step of the *i*-th candidate DOA in the *l*-th particle. After the position update, the particle is constrained within the LOD to ensure that all candidate angles remain in the local search region, which can be expressed as

(44) $${\hat{\theta }}_{l}^{\prime k+1}=\Pi_{\mathrm{LOD}}\left({\hat{\theta }}_{l}^{\prime k+1}\right),\;$$

where $\Pi_{\mathrm{LOD}}\left(\cdot \right)$ denotes the projection or boundary constraint operator onto the LOD. This operation prevents particles from moving outside the local angular domain and guarantees that the optimization process is performed only within the constructed LOD.

By incorporating the first two contributions proposed in this paper, the overall positioning algorithm procedure is summarized in Algorithm 1. Where $K_{max}$ denotes the maximum number of iterations, and *k* denotes the current iteration number. $\tilde{P}$ denotes the number of initialized particles.
**Algorithm 1:** Overall optimization model
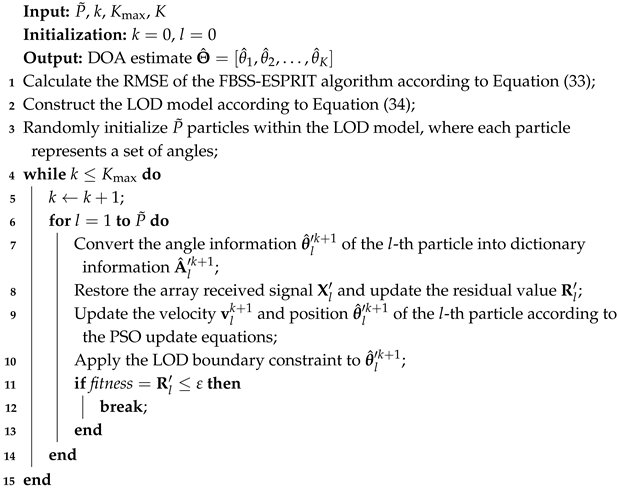



In Algorithm 1, Steps 1–2 are devoted to the construction of the LOD. Step 3 randomly initializes $\tilde{P}$ particles within the LOD model, where each particle carries a set of angular information serving as the initial input for PSO iteration. Steps 4–12 constitute the iterative process of signal reconstruction using the PSO algorithm. The velocity and position update rules of the PSO algorithm can be found in reference [14]. From the above steps, it can be observed that the initial solution space of the algorithm is significantly reduced, and the DOA estimation accuracy is improved through the enhanced signal reconstruction method.

## 5. Results

In this section, the proposed LOD-PSO algorithm is compared with MSCM-OMP [41], FOMP [39], FBSS-ESPRIT [25], MESA [30], MUC-WSF [12], ss-MUSIC [23], and MARS [42] through multiple sets of experiments. Among these methods, ss-MUSIC [23] and FBSS-ESPRIT [25] are subspace-decomposition-based algorithms, MESA [30] and MUC-WSF [12] are subspace-fitting-based algorithms, and MSCM-OMP [41], FOMP [39], and MARS [42] are CS-based algorithms. By comparing the LOD-PSO algorithm with different types of algorithms, the general applicability of the proposed algorithm is demonstrated.

A uniform linear array is considered. The parameters are set as M=15 , K=2 , and Q=5 . In addition, the distance *d* between two adjacent sensors is set to one half of the signal wavelength λ , i.e., d=λ/2 . The angular search step size of all algorithms is set to $0.01^{\circ }$ . All results are obtained from 100 Monte Carlo (MC) trials. The RMSE is used to evaluate the estimation accuracy and is defined as in (33). All experiments are conducted utilizing MATLAB R2023b on a personal computer with an Intel R7-4800H CPU and 16 GB RAM, without GPU acceleration. The per-frame processing latency of all methods is measured on this identical platform using the same input data. The timing covers the complete pipeline from the array received data to the output localization estimate, and, for LOD-PSO, it includes its FBSS-ESPRIT coarse-estimation stage. A warm-up run is performed in advance to eliminate the first-call overhead, thereby ensuring a fair comparison among all algorithms under identical conditions.

The parameters of the PSO stage are configured as follows: the swarm size is set to $\tilde{P}=30$ and the maximum number of iterations to $T_{p}=50$ , while the inertia weight ω decreases linearly from $\omega_{\max}=0.9$ to $\omega_{\min}=0.4$ . Since the particle swarm is confined to a LOD centered on the FBSS-ESPRIT coarse estimate, such a small swarm size and iteration number are sufficient for stable convergence, which is one of the reasons for the low latency of LOD-PSO. Under the hovering condition, the position jitter of each UAV is modeled as a zero-mean, isotropic, temporally white Gaussian process. Given a hovering oscillation velocity of $v_{\mathrm{osc}}=8.99\;m/s$ and a per-frame coherent processing time of $T_{s}\approx 5.6\;\mathrm{ms}$ as the characteristic integration interval of the jitter, the displacement standard deviation of each UAV is $\sigma_{p}=v_{\mathrm{osc}}\cdot T_{s}=8.99\times 5.6\times 10^{-3}\approx 0.05\;m$ , and the corresponding jitter variance is $\sigma_{\mathrm{osc},\;k}^{2}=\sigma_{p}^{2}\approx 2.5\times 10^{-3}\;m^{2}$ ( k=1, 2, 3 , identical for all three UAVs).

### 5.1. DOA Estimation Accuracy

#### 5.1.1. Angular Distribution of DOA Estimates

**Figure 7:**
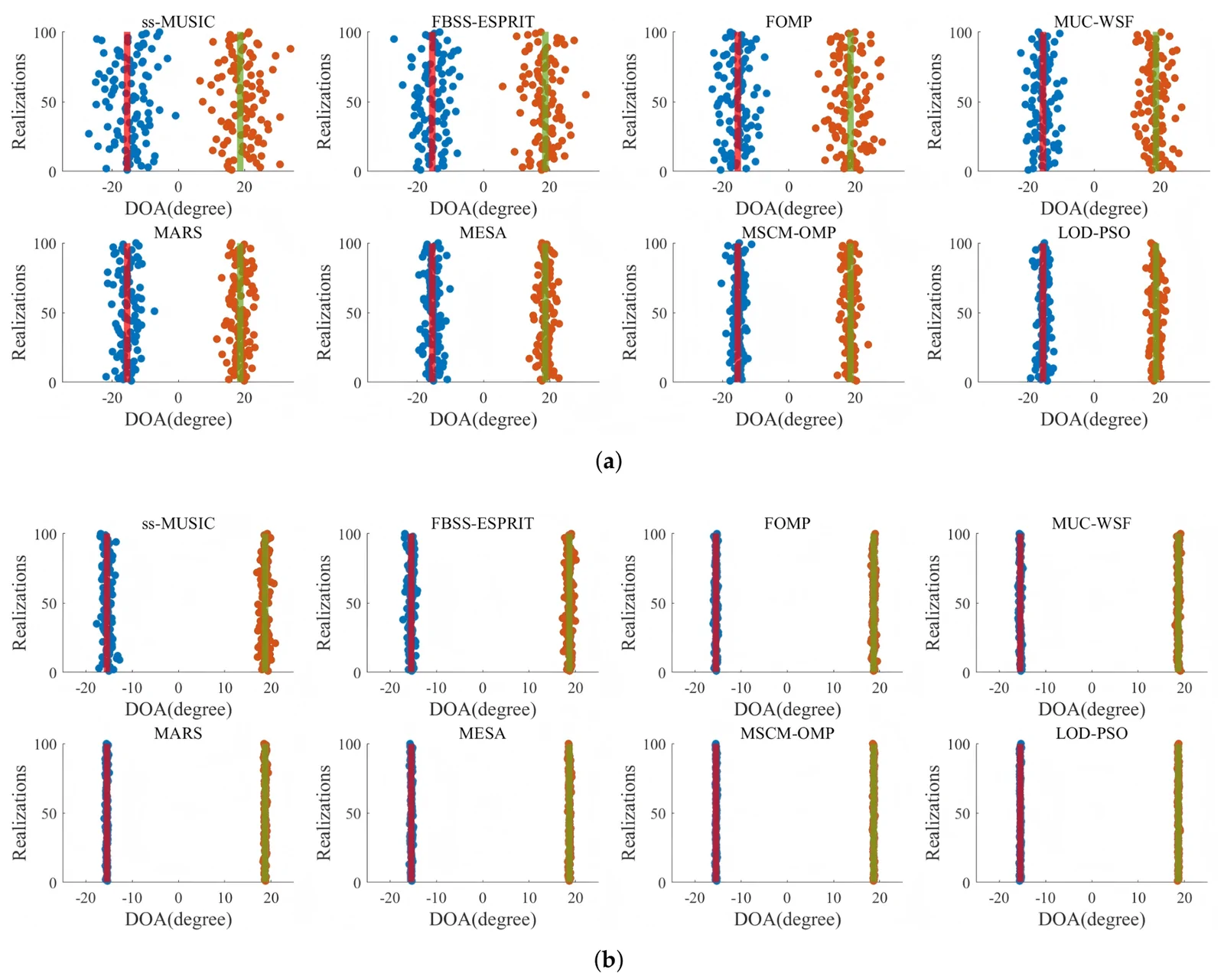
Angular distributions of DOA estimates obtained by different algorithms. MC = 100, and the randomly selected DOAs are $\left[-15.43^{\circ },\;18.71^{\circ }\right]$ . (**a**) SNR = −5 dB. (**b**) SNR = 2 dB.

Figure 7 shows the angular distributions of DOA estimates obtained by different algorithms under incoherent signal conditions. The true DOAs are randomly generated as $\left[-15.43^{\circ },\;18.71^{\circ }\right]$ , and 100 Monte Carlo (MC) trials are conducted. Figure 7a presents the angular distributions of different algorithms when the SNR is −5 dB, whereas Figure 7b presents the corresponding results when the SNR is 2 dB.

As shown in Figure 7a, when the SNR is −5 dB, the DOA estimates obtained by MSCM-OMP [41], FOMP [39], FBSS-ESPRIT [25], MESA [30], MUC-WSF [12], ss-MUSIC [23], and MARS [42] exhibit relatively large angular fluctuations around the two true DOAs. In particular, the estimated angles of several comparison algorithms are widely scattered, indicating that their estimation performance is significantly affected by noise in low-SNR scenarios. This phenomenon is especially evident near the two target angles, where the estimated DOAs deviate noticeably from the ground-truth values and the distribution of the estimation points becomes dispersed. The large dispersion reflects the reduced stability and robustness of these algorithms under severe noise interference.

In contrast, the proposed LOD-PSO algorithm yields DOA estimates that are tightly concentrated around the true DOAs even at −5 dB. The estimated angle distributions corresponding to the two sources show narrow spreads and remain close to $-15.43^{\circ }$ and $18.71^{\circ }$ , respectively. This demonstrates that the proposed algorithm can effectively suppress the influence of noise and maintain high estimation accuracy under low-SNR conditions. Compared with the other algorithms, LOD-PSO exhibits better robustness and stronger global search capability, thereby reducing the probability of large angular estimation errors.

When the SNR increases to 2 dB, as shown in Figure 7b, the angular distributions of all algorithms become more concentrated around the true DOAs, indicating that the improvement in SNR enhances the DOA estimation accuracy. However, differences among the algorithms can still be observed. Although the estimation fluctuations of the comparison algorithms are reduced compared with those at −5 dB, several methods still present a certain degree of angular dispersion. By contrast, the proposed LOD-PSO algorithm produces the most compact distributions around the two true DOAs, with almost all estimated angles located near the reference lines. This indicates that LOD-PSO not only performs well in low-SNR environments but also maintains superior estimation stability when the signal quality improves.

#### 5.1.2. RMSE vs. SNR

**Figure 8:**
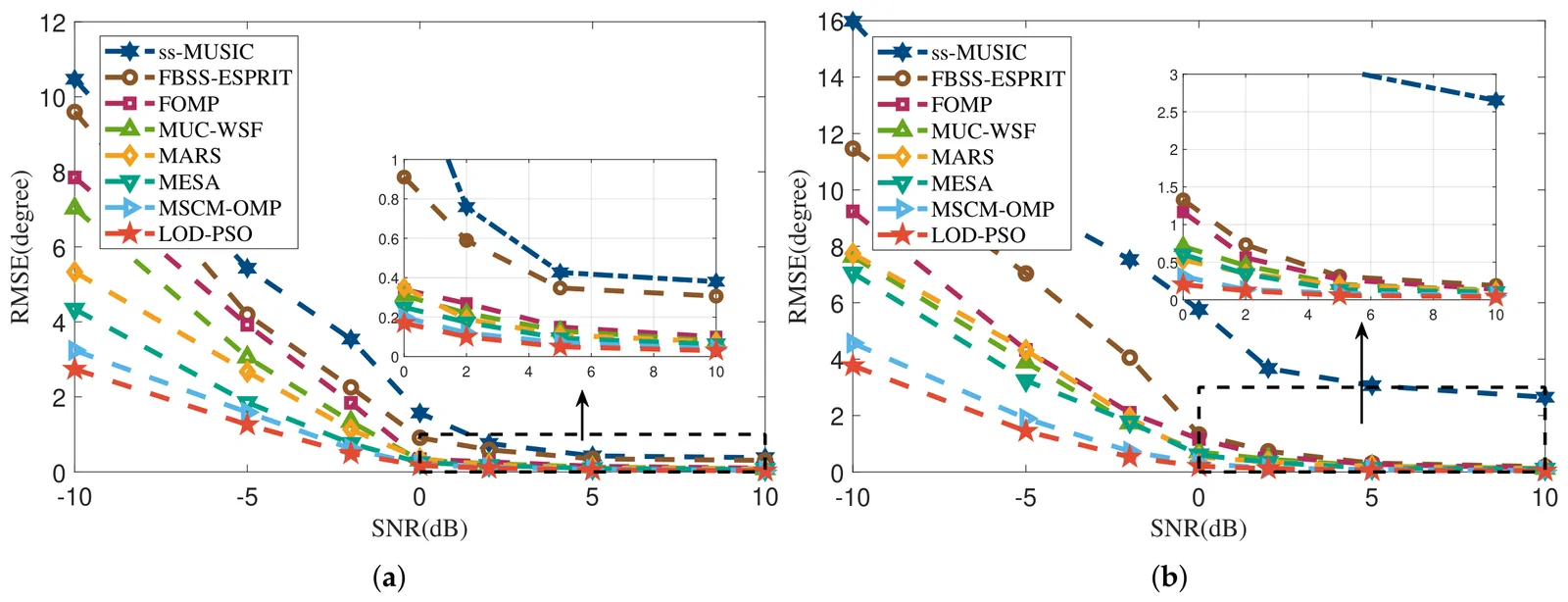
Comparison of RMSE versus SNR for different algorithms: (**a**) Incoherent signals. (**b**) Coherent signals.

Figure 8 presents the comparison of RMSE versus SNR for different algorithms under incoherent signal conditions, as shown in Figure 8a, and coherent signal conditions, as shown in Figure 8b. It can be observed that the RMSE values of all algorithms decrease with increasing SNR, indicating that the DOA estimation accuracy improves as the noise level decreases. However, the proposed LOD-PSO algorithm consistently achieves the lowest RMSE over the entire SNR range in both scenarios.

For incoherent signals, as shown in Figure 8a, MSCM-OMP [41], FOMP [39], and FBSS-ESPRIT [25] exhibit relatively large DOA estimation errors in the low-SNR region. In particular, when SNR=−5 dB, their RMSE values are $1.59^{\circ }$ , $3.92^{\circ }$ , and $4.20^{\circ }$ , respectively, whereas the RMSE of the proposed LOD-PSO algorithm is only $1.26^{\circ }$ . Thus, the RMSE of LOD-PSO is 79.25% , 32.14% , and 30.00% of that achieved by MSCM-OMP [41], FOMP [39], and FBSS-ESPRIT [25], respectively. This indicates that the proposed algorithm can effectively suppress noise-induced estimation errors and provide more accurate DOA estimates under low-SNR conditions.

For coherent signals, as shown in Figure 8b, the estimation accuracy of most algorithms deteriorates due to the correlation among sources. This degradation is particularly significant for OMP-derivative algorithms and conventional subspace-based methods, since source coherence may cause covariance matrix rank deficiency and inaccurate direction selection. At SNR=−5 dB, the RMSE values of MSCM-OMP [41], FOMP [39], and FBSS-ESPRIT [25] are $1.89^{\circ }$ , $4.33^{\circ }$ , and $7.04^{\circ }$ , respectively, while LOD-PSO achieves an RMSE of $1.45^{\circ }$ . Therefore, the RMSE of LOD-PSO is 76.72% , 33.49% , and 20.60% of that obtained by MSCM-OMP [41], FOMP [39], and FBSS-ESPRIT [25], respectively. These results demonstrate that LOD-PSO can effectively alleviate the negative effects of source correlation and noise.

Moreover, the enlarged regions in Figure 8 further show that LOD-PSO maintains a clear advantage in the medium- and high-SNR ranges. Although the RMSE values of all algorithms gradually decrease as the SNR increases, LOD-PSO still provides the smallest estimation errors. Overall, the proposed LOD-PSO algorithm consistently outperforms the existing state-of-the-art algorithms in terms of DOA estimation performance. The improvement is especially significant under low-SNR and coherent-source conditions, demonstrating the robustness, accuracy, and adaptability of the proposed method.

### 5.2. Real-Time Performance Comparison of Different Algorithms

Table 1 presents the computational complexity comparison of the eight algorithms considered in this article, and Table 2 summarizes the parameters involved in the analysis. Throughout the following discussion, we treat the array size *M*, the grid size *L*, the dictionary size $L_{c}$ , and the snapshot number *Q* as the growing dimensions, whereas the source number *K*, the swarm size $\tilde{P}$ , and the iteration counts ( $T_{p}$ , $T_{m}$ , $T_{w}$ ) are regarded as bounded constants, as is standard in asymptotic complexity analysis. Under the experimental configuration M=15 , K=2 , and Q=5 , we have M>Q , so that the eigendecomposition term $O(M^{3})$ dominates the covariance-formation term $O(M^{2}Q)$ . The computational cost of each algorithm can be decomposed into two parts: *covariance/subspace construction* and *parameter search/iteration*.

#### 5.2.1. Covariance/Subspace Construction

The sample covariance matrix $\hat{R}$ is shared by ss-MUSIC, MSCM-OMP, FBSS-ESPRIT, MESA, MUC-WSF, the proposed LOD-PSO, and its formation costs $O(M^{2}Q)$ . Among these, ss-MUSIC, FBSS-ESPRIT, and MUC-WSF must additionally perform an eigendecomposition of $\hat{R}$ (or of the forward–backward smoothed covariance) at a cost of $O(M^{3})$ to extract the signal/noise subspaces $V_{S}$ and $V_{N}$ , giving a construction overhead of $O(M^{2}Q+M^{3})$ . MSCM-OMP and MESA operate directly on the covariance and thus incur only $O(M^{2}Q)$ , whereas FOMP works on the raw snapshots and MARS processes a single-snapshot statistic, so their construction cost is negligible, i.e., O(MQ) . Crucially, the proposed LOD-PSO evaluates the deterministic maximum-likelihood (SML) criterion directly on $\hat{R}$ and never forms nor inverts an M×M matrix; hence its construction overhead is only $O(M^{2}Q)$ , strictly cheaper than the eigendecomposition-based subspace methods.

#### 5.2.2. Parameter Search/Iteration

For ss-MUSIC, the dominant cost is the exhaustive traversal of the spatial spectrum, O(LM(M−K)) , which grows linearly with the grid size *L* and becomes the bottleneck once the angular resolution is tightened to $0.01^{\circ }$ . MSCM-OMP and FOMP rely on greedy sparse reconstruction over an overcomplete dictionary, costing $O(K\;L_{c}\;M^{2})$ and $O(K\;L_{c}\;M\;Q)$ , respectively, whose dominant factor is the dictionary size $L_{c}$ rather than *M*; to guarantee angular resolution, $L_{c}$ must be kept large, which keeps their complexity high. FBSS-ESPRIT avoids any grid search and only solves the rotational-invariance equation, at a cost of $O(MK^{2}+K^{3})$ ; its overall complexity is therefore governed by the $O(M^{3})$ eigendecomposition. MESA is dominated by the sequential ADMM outer loop, $O(T_{m}\;K\;M^{3})$ , which cannot be parallelized. MUC-WSF performs a nonlinear weighted-subspace-fitting search, $O(T_{w}\left(M^{3}+M^{2}K\right))$ . MARS employs OMP over a temporally correlated reduced search space of size $L_{r}\ll L$ , giving $O(K\;L_{r}\;M^{2})$ ; although far cheaper than the full-grid ss-MUSIC, it still scales with the reduced grid size $L_{r}$ .

For the proposed LOD-PSO, the parameter-search cost is that of the particle swarm confined to the limited optimization domain (LOD). Each fitness evaluation forms the array manifold $A\left(\theta \right)\in C^{M\times K}$ and the orthogonal projector $P_{A}^{⊥}=I-A{\left(A^{H}A\right)}^{-1}A^{H}$ , and it then evaluates $\mathrm{tr}(P_{A}^{⊥}\hat{R})$ ; since the only inversion is of the K×K matrix $A^{H}A$ , the per-evaluation cost is $O(M^{2}K+MK^{2}+K^{3})$ , with *no* $O(M^{3})$ term. With $\tilde{P}$ particles evolved over $T_{p}$ iterations, the parameter-search complexity is $O(\tilde{P}T_{p}\left(M^{2}K+MK^{2}+K^{3}\right))$ . Because the LOD, extracted from a low-cost coarse localization pass, confines the swarm to only a small fraction of the full field of view $\left[-90^{\circ },\;90^{\circ }\right]$ , the seeding of the particles takes only $O(\tilde{P})$ scatter operations and the product $\tilde{P}T_{p}$ behaves as a bounded small constant; consequently, $O\left(\tilde{P}T_{p}\left(M^{2}K+MK^{2}+K^{3}\right)\right)\ll O\left(L\;M\left(M-K\right)\right)$ for any practically useful angular resolution.

#### 5.2.3. Overall Comparison

As can be seen from Table 1, the proposed LOD-PSO attains a highly competitive computational complexity among all the compared algorithms and in particular delivers the lowest *measured* single-frame latency among all *accurate* super-resolution estimators. This is due to two reasons: (i) the limited optimization domain restricts the parameter search to a very small feasible region, so that $\tilde{P}T_{p}$ acts as a bounded small constant; and (ii) LOD-PSO evaluates the SML criterion directly on the sample covariance, so that its refinement stage scales only as $O(\tilde{P}T_{p}\left(M^{2}K+MK^{2}+K^{3}\right))$ and never invokes a full-resolution grid or dictionary traversal. Asymptotically, treating *K*, $\tilde{P}$ , and $T_{p}$ as constants, the refinement cost of LOD-PSO reduces to $O(M^{2}Q)$ ; however, as detailed below, its *overall* complexity still inherits the $O(M^{3})$ term of the coarse-initialization stage.

For completeness, the overall complexity of LOD-PSO must include its coarse-initialization stage. LOD-PSO first runs FBSS-ESPRIT to obtain the dictionary centers at a cost of $O(M^{2}Q+M^{3})$ , and it subsequently performs the swarm refinement at $O(\tilde{P}T_{p}\left(M^{2}K+MK^{2}+K^{3}\right))$ ; its total cost is therefore $O\left(M^{2}Q+M^{3}\right)+O\left(\tilde{P}T_{p}\left(M^{2}K+MK^{2}+K^{3}\right)\right)$ . It follows that LOD-PSO shares the same $O(M^{3})$ eigendecomposition lower bound as the subspace-based methods, rather than lying strictly below it. Its practical advantage does not stem from avoiding the eigendecomposition, but from replacing the expensive fine-resolution search with a swarm search confined to the narrow LOD, for which $\tilde{P}T_{p}$ behaves as a bounded small constant. This is precisely why, at the $0.01^{\circ }$ resolution adopted throughout, the measured single-frame latency of LOD-PSO is significantly lower than that of the grid/dictionary-heavy methods.

In contrast, ss-MUSIC, FBSS-ESPRIT, and MUC-WSF all retain the $O(M^{3})$ eigendecomposition (which dominates $O(M^{2}Q)$ since M>Q ); MESA carries the sequential $O(T_{m}KM^{3})$ ADMM cost; MSCM-OMP, FOMP, and MARS remain governed by the dictionary/grid factors $L_{c}$ or $L_{r}$ ; and ss-MUSIC additionally suffers from the O(LM(M−K)) spectral traversal. Grouping these observations by dominating order, the sparse-recovery methods MARS, MSCM-OMP, and FOMP scale with the reduced grid or dictionary size ( $L_{r}$ or $L_{c}$ ), whereas the eigendecomposition-based methods LOD-PSO, FBSS-ESPRIT, MESA, and MUC-WSF, together with the full-grid ss-MUSIC, are all bounded below by $O(M^{3})$ ; among the latter, LOD-PSO and FBSS-ESPRIT remain the cheapest because their post-eigendecomposition stages contribute only a bounded constant, while MESA, MUC-WSF, and ss-MUSIC pay heavy iterative or full-grid overheads on top of the same $O(M^{3})$ term. Where FBSS-ESPRIT is the fastest owing to its closed-form estimator on the small array M=15 , LOD-PSO follows immediately because its swarm refinement adds only a bounded small constant on top of the same $O(M^{3})$ initialization, the placement of MARS ahead of MSCM-OMP follows from the typical regime $L_{r}<L_{c}$ , and ss-MUSIC ranks slowest because its full-grid spectral traversal O(LM(M−K)) explodes at the $0.01^{\circ }$ resolution on top of the $O(M^{3})$ eigendecomposition. LOD-PSO is therefore the only algorithm that pairs a subspace-grade $O(M^{3})$ lower bound with a bounded-constant refinement cost, while still delivering maximum-likelihood-grade super-resolution accuracy.

### 5.3. Ablation Study

To attribute the observed performance gains to each individual contribution, we conduct an ablation study that isolates the effect of the three interrelated components proposed in this paper: the displacement-aware reception model, the LOD, and the PSO-based joint reconstruction. Starting from a common baseline—uniform overcomplete dictionary OMP reconstruction built on a stationary-UAV reception model (denoted UOD + OMP)—we activate the three contributions one at a time and evaluate the resulting DOA root-mean-square error (RMSE) and the per-frame processing latency. To make the ablation consistent with the main claims of this paper, we consider two representative cases. The first case corresponds to non-coherent sources at SNR=2dB , which evaluates the individual contribution of each component under a moderate-SNR non-coherent condition. The second case corresponds to coherent sources at SNR=−5dB , which is a more challenging low-SNR multipath scenario and directly reflects the regime where the proposed LOD-PSO method is expected to provide its main advantage. In both cases, every other array, channel, snapshot, and jitter parameter is held identical to the common baseline so that any change in performance can be ascribed solely to the newly activated component.

The ablation reveals that the three contributions are complementary and address distinct error mechanisms rather than overlapping in function. For the non-coherent case in Table 3, progressing from B0 to B1, the introduction of the displacement-aware reception model reduces the DOA RMSE from $1.235^{\circ }$ to $0.843^{\circ }$ while leaving the latency almost unchanged (from 0.321s to 0.303s ). This is expected: the displacement-aware model does not alter the estimator or its search space but instead removes the transceiver geometric mismatch introduced by UAV hovering displacement, thereby suppressing the motion-induced component of the estimation error. In the notation of our error-propagation framework, it acts primarily on $\Delta \tilde{\epsilon }$ .

Progressing from B1 to B2, activating the LOD—while still employing the greedy OMP solver—reduces the per-frame latency by roughly an order of magnitude, from 0.303s to 0.031s . This dramatic reduction arises because the LOD confines the reconstruction to a compact, statistically bounded search window centered on the FBSS-ESPRIT estimate, rather than scanning the full uniform grid. Notably, the DOA RMSE also improves from $0.843^{\circ }$ to $0.231^{\circ }$ , indicating that pruning implausible grid atoms not only accelerates the solver but also removes spurious candidates that would otherwise degrade the estimate. The LOD therefore acts principally on the latency term, and thereby—through the algorithmic delay Δt —indirectly on $\Delta \tilde{\epsilon }$ .

Finally, progressing from B2 to B3, replacing OMP with the joint PSO reconstruction yields a further improvement in DOA accuracy under non-coherent sources, driving the DOA RMSE down from $0.231^{\circ }$ to $0.100^{\circ }$ , with a further slight reduction in latency (from 0.031s to 0.023s ). This confirms that the PSO reconstruction refines the sparse solution beyond the reach of the greedy OMP by jointly optimizing the on-grid atoms, thereby mitigating the residual grid-mismatch and multipath-induced errors that persist even in the non-coherent regime, operating directly on the reconstruction-induced error term $\Delta \tilde{\varepsilon }$ .

The additional low-SNR coherent-source ablation in Table 4 further confirms the necessity of the proposed components in the most challenging operating regime. Compared with the non-coherent case, the baseline C0 suffers a much larger RMSE of $6.742^{\circ }$ , because source coherence causes covariance-rank degradation and also aggravates the sequential error-propagation problem of OMP. After introducing the displacement-aware reception model, the RMSE decreases from $6.742^{\circ }$ to $5.618^{\circ }$ , while the latency changes only slightly from 0.326s to 0.309s . This indicates that the displacement-aware model remains effective under coherent low-SNR conditions, but its role is mainly to correct the UAV-motion-induced geometric mismatch rather than to resolve the source-correlation problem.

When the LOD is further activated, the latency is reduced from 0.309s to 0.059s , demonstrating that the LOD is the principal contributor to real-time performance even under low-SNR coherent-source conditions. Meanwhile, the RMSE decreases from $5.618^{\circ }$ to $2.387^{\circ }$ . This improvement is more pronounced than in the non-coherent case because the global UOD contains many highly correlated and noise-sensitive atoms under low-SNR coherent conditions. By restricting the reconstruction to an SNR-adaptive local window centered on the FBSS-ESPRIT estimate, the LOD removes a large number of implausible atoms and therefore reduces the probability that OMP selects spurious directions.

Finally, replacing OMP with PSO in the LOD gives a further RMSE reduction from $2.387^{\circ }$ to $1.450^{\circ }$ , while the latency is further reduced from 0.059s to 0.041s . This additional gain is particularly important for coherent sources. Unlike OMP, which estimates the sources sequentially and therefore suffers from residual-coupling and error-cascade effects, the PSO-based reconstruction treats the multi-source DOA vector as a single particle state and jointly optimizes all source directions. Therefore, it directly mitigates the coherent-source coupling that remains after the displacement-aware modeling and LOD pruning stages. This result explains why the full LOD-PSO method achieves its largest relative advantage in the low-SNR coherent-source regime.

In summary, the ablation study demonstrates that each contribution plays a distinct and mutually reinforcing role: the displacement-aware reception model targets the geometric mismatch term $\Delta \tilde{\epsilon }$ , the LOD targets the latency and, through Δt , further reduces $\Delta \tilde{\epsilon }$ , and the PSO reconstruction targets the residual reconstruction error $\Delta \tilde{\varepsilon }$ . The newly added coherent-source ablation at SNR=−5dB further verifies that this division of function still holds under the low-SNR coherent condition emphasized in this paper. In particular, the PSO reconstruction contributes a larger relative accuracy gain in the coherent case than in the non-coherent case, confirming that the proposed joint reconstruction is essential for breaking the source-correlation cascade of greedy OMP. It is their combination in the full LOD-PSO method that attains simultaneously the lowest DOA RMSE and the lowest latency in both tested ablation scenarios.

### 5.4. Positioning Error

The *O* agricultural machinery units are randomly positioned within the UAV coverage region, forming the set $V=[V_{1},\;V_{2},\;\ldots ,\;V_{O}]$ . In the UAV reference frame, the positions of UAVs $U_{D1}$ , $U_{D2}$ , and $U_{D3}$ are assumed to be [0, 700m] , [0, 0] , and [700m, 0] , respectively, and at least one agricultural machinery unit within the UAV coverage region has available GPS positioning information during the localization process.

In the experiment, the signal sources are coherent, O=100 , and the SNR is set to 2dB and 10dB . The proposed LOD-PSO algorithm is compared against seven representative baselines spanning the three mainstream technical routes: the subspace-decomposition methods ss-MUSIC [23] and FBSS-ESPRIT [25], the subspace-fitting methods MESA [30] and MUC-WSF [12], and the compressed-sensing methods MSCM-OMP [41], FOMP [39], and MARS [42]. Each algorithm first performs DOA estimation for the *O* machinery positions to obtain the azimuth information ${\hat{\varphi }}_{k}$ , from which the two-dimensional position $\hat{p}$ is recovered by the least-squares cross-fix of Equation (7). Utilizing these angular estimates, the total average positioning error $\Delta \tilde{\sigma }$ over *O* trials is computed as

(45) $$\begin{array}{c} \Delta \tilde{\sigma }=\sqrt{\frac{1}{J\times O}\sum_{j=1}^{O}\sum_{i=1}^{J}{\left({\tilde{x}}_{j,\;i}-x_{j}\right)}^{2}+{\left({\tilde{y}}_{j,\;i}-y_{j}\right)}^{2}},\; \end{array}$$

where $x_{j}$ and $y_{j}$ denote the true *x*- and *y*-coordinate positions of the *j*th agricultural machinery in the UAV coordinate system, and ${\tilde{x}}_{j,\;i}$ and ${\tilde{y}}_{j,\;i}$ represent the corresponding estimated positions of the *j*th machinery in the *i*th trial.

Consistent with the mean-square error decomposition of Equation (14), the empirical total positioning error is the superposition of two mutually independent components, $\Delta \tilde{\sigma }=\sqrt{\Delta {\tilde{\varepsilon }}^{2}+\Delta {\tilde{\epsilon }}^{2}}$ . Here, $\Delta \tilde{\varepsilon }$ is the *DOA-estimation error* corresponding to the accuracy term of Equation (11), which is governed by the angular RMSE together with the geometric configuration (the GDOP of Equation (8)); it is measured under the ideal stationary-UAV setting and after the proposed displacement-aware reception model (Equations (19)–(23)) has removed the platform-drift contribution. In contrast, $\Delta \tilde{\epsilon }$ is the UAV-oscillation error corresponding to the real-time term of Equation (13), which grows quadratically with the reporting latency *t* (i.e., Δt ) and reflects the platform jitter accumulated during processing. Table 5 reports, for each algorithm, the DOA-estimation error $\Delta \tilde{\varepsilon }$ , the single-frame processing latency *t*, the isolated oscillation-induced error $\Delta \tilde{\epsilon }$ , and the resulting total error $\Delta \tilde{\sigma }$ .

**DOA-estimation error $\Delta \tilde{\varepsilon }$ .** The proposed LOD-PSO consistently attains the smallest DOA-induced error at both SNR levels ( 0.5345m at 2dB and 0.3223m at 10dB ). This directly follows from the accuracy term of Equation (11): by refining the angular resolution within the local overcomplete dictionary and jointly optimizing the multi-source angle vector through the particle swarm, LOD-PSO delivers the lowest RMSE among all methods, so its efficiency factor approaches the ideal limit $\eta_{k}\to 1$ . By contrast, the subspace-decomposition methods FBSS-ESPRIT and ss-MUSIC suffer from covariance rank deficiency under coherent sources and yield the largest values ( 6.0645m and 4.8752m at 2dB ), and the greedy FOMP is degraded by the source-correlation cascade; MESA, MUC-WSF, and MARS occupy an intermediate tier, with MSCM-OMP being the strongest baseline yet still inferior to LOD-PSO.

**UAV-oscillation error $\Delta \tilde{\epsilon }$ and latency** ***t*****.** During the interval between the direction-finding instant and the reporting instant, each UAV drifts at an approximately constant operating speed ∥v∥ , so the oscillation-induced displacement error is governed solely by the algorithm’s processing latency, $\Delta \tilde{\epsilon }=∥v∥\;t$ (Equations (13) and (14)). Consequently, $\Delta \tilde{\epsilon }$ increases *monotonically* with *t*: the shorter the latency, the smaller the drift. The closed-form FBSS-ESPRIT, being the fastest estimator for the small array M=15 , therefore incurs the *smallest* oscillation error ( 0.0477m / 0.0441m ), immediately followed by LOD-PSO ( 0.2087m / 0.0945m ), whose complexity is the lowest among all *accurate* super-resolution estimators (Equation (40)). At the opposite extreme, the dictionary/grid/iteration-heavy methods pay a heavy price: the full-grid ss-MUSIC ( 0.5910s ), the nonlinear-search MUC-WSF ( 0.4210s ), and the ADMM-based MESA ( 0.3560s ) allow the UAVs to drift appreciably, producing the largest oscillation errors ( 5.3166m , 3.7873m , and 3.2026m at 2dB ); the large-dictionary FOMP ( 0.2480s ) follows the same trend. This behavior is now fully consistent with the model: latency and oscillation error rise and fall together for every method, without exception.

**Total error $\Delta \tilde{\sigma }$ .** The total positioning error combines the two orthogonal contributions through their root-mean-square, $\Delta \tilde{\sigma }=\sqrt{\Delta {\tilde{\varepsilon }}^{2}+\Delta {\tilde{\epsilon }}^{2}}$ , rather than a plain summation, so that the angular-estimation error and the UAV-oscillation error are aggregated in the mean-square sense consistent with their statistically independent origins. Under this metric, LOD-PSO achieves the lowest total positioning error in every configuration ( 0.5738m at 2dB and 0.3359m at 10dB ). At SNR=10dB , its total error is 30.36% , 12.95% , 16.97% , 6.20% , 10.61% , 9.01% , and 47.31% of that of MSCM-OMP, FOMP, FBSS-ESPRIT, ss-MUSIC, MESA, MUC-WSF, and MARS, respectively; the corresponding ratios at 2dB are 46.44% , 12.56% , 9.46% , 7.95% , 17.02% , 14.06% , and 52.37% , showing that the advantage is most pronounced at low SNR, where the DOA error is most severe. Because LOD-PSO provides the smallest total positioning error while maintaining a short latency, it achieves a favorable balance between localization accuracy and real-time efficiency in Equation (14). It should also be noted that the practical applicability of the obtained accuracy depends on both the SNR condition and the required precision of the agricultural operation. For example, at SNR=2dB , the total error of LOD-PSO is 0.5738m , which is more suitable for agricultural UAV tasks with moderate positioning requirements, such as field inspection, crop-growth monitoring, wide-area mapping, and general navigation assistance. In contrast, under a more favorable signal condition such as SNR=10dB , the total error is reduced to 0.3359m , making the method more applicable to higher-precision tasks such as seeding, plant protection, and precision spraying. Therefore, rather than claiming that all sub-meter errors are sufficient for operations requiring 10– 50cm accuracy, the revised interpretation qualifies the applicability of LOD-PSO according to the operating SNR and the target agricultural operation. Combined with a latency compatible with the real-time response requirement of farm equipment, these results indicate that LOD-PSO provides the best overall accuracy, robustness, and real-time performance for cooperative multi-UAV localization in smart-tillage scenarios.

All results reported in this section are obtained from N=100 independent Monte Carlo trials at each SNR point (SNR = signal-to-noise ratio, in dB). In each trial, the source angles (the true directions-of-arrival of the UAV emitter), the noise realizations (the independently generated measurement-noise samples), and the platform-jitter displacements (the small random offsets of the agricultural machinery platform) are redrawn independently so that the *N* trials are mutually independent. To make the statistical uncertainty of these estimates explicit, we additionally characterize their trial-to-trial variability. For a second-order statistic such as the RMSE, the estimator $\hat{\mathrm{RMSE}}=\sqrt{\frac{1}{N}\sum_{i}{∥{\hat{p}}_{i}-p∥}^{2}}$ , where $\hat{\mathrm{RMSE}}$ is the estimated root-mean-square error, ${\hat{p}}_{i}$ is the estimated position vector in trial *i*, p is the true position vector, and ${∥{\hat{p}}_{i}-p∥}^{2}$ is the squared localization error of trial *i*, has, by the delta method (a first-order error-propagation technique), a coefficient of variation $c_{v}=\frac{1}{2}\sqrt{(v/\mu^{2})/N}$ , where $c_{v}$ is the relative standard deviation of the RMSE estimate, μ and *v* denote the mean and variance of the per-trial squared error $x_{i}={∥{\hat{p}}_{i}-p∥}^{2}$ , and *N* is the trial count. Under the standard zero-mean Gaussian-error approximation ( $v/\mu^{2}=2$ , i.e., the squared error follows an approximate chi-square distribution so that its variance equals twice the square of its mean), this reduces to the conservative bound

(46) $$c_{v}=\frac{1}{\sqrt{2N}}\;\overset{\;N=100\;}{\to }\;7.1\%,\;$$

so that the corresponding 95% confidence interval (the interval covering the true value with 95% probability, using the normal critical value 1.96 ) of every reported RMSE value has a relative half-width of $1.96\;c_{v}=1.96/\sqrt{2N}\approx \pm 13.9\%$ (i.e., each RMSE should be read as $\hat{\mathrm{RMSE}}\times \left(1\pm 0.139\right)$ ). Because the performance separations in Table 5 are far larger than this interval—for instance, the 91% gap between LOD-PSO ( 0.5738m , the proposed method’s total error at 2dB ) and the next-best MARS ( 1.0957m , the smallest error among competing methods at the same 2dB ), whose 95% intervals [0.49, 0.65]m (computed as 0.5738×(1±0.139) ) and [0.94, 1.25]m (computed as 1.0957×(1±0.139) ) do not overlap—the confidence intervals of the compared methods are mutually disjoint (non-overlapping) and the method ranking is statistically unambiguous (clearly distinguishable) at the present sample size (the current N=100 ). Increasing *N* would narrow these intervals further but would neither alter the ranking nor affect any conclusion of this work.

## 6. Discussion

The simulation results reported in Section 5 can be coherently interpreted only when they are read against the error-decomposition theory established in Section 2. Equation (14) predicts that the expected total mean-square localization error separates into a configuration- and accuracy-governed term $\sum_{k}\mathrm{CRLB}\left(\varphi_{k}\right)/\eta_{k}\;{∥\partial \hat{p}/\partial \varphi_{k}∥}^{2}$ and a latency-governed term $\sum_{k}\sigma_{\mathrm{osc},\;k}^{2}R_{k}^{-2}\Delta t^{2}$ , and that these two terms constitute an intrinsic trade-off rather than two independently optimizable quantities. The central empirical finding of this work is that LOD-PSO is the only method among the eight compared that suppresses both terms simultaneously: it attains the smallest DOA-induced error $\Delta \tilde{\varepsilon }$ (Table 5, 0.5345m at 2dB ), thereby driving the efficiency factor $\eta_{k}$ toward its ideal limit, while its grid/dictionary-free complexity $O(M^{2}Q+M^{3})$ keeps its latency *t* among the lowest of all methods ( 0.0232s at 2dB , second only to FBSS-ESPRIT) and hence keeps its oscillation-induced error $\Delta \tilde{\epsilon }$ correspondingly small ( 0.2087m ). No other method holds both terms small at once: the fastest estimator, FBSS-ESPRIT, attains the smallest oscillation error ( 0.0477m ) only at the price of by far the largest accuracy error, whereas the more accurate spectral and sparse baselines incur latencies that inflate their oscillation term, so that LOD-PSO alone reaches the smallest total error $\Delta \tilde{\sigma }$ ( 0.5738m ).

A closer reading of Table 5 exposes a failure mode that a purely latency-oriented analysis would overlook, and that only the decomposition of Equation (14) renders visible. FBSS-ESPRIT records the lowest wall-clock latency of all methods ( 0.0053s at 2dB ) and, as a direct consequence, the *smallest* oscillation-induced error ( 0.0477m ); yet it produces by far the *largest* DOA-induced error ( 6.0645m ) so that its total error ( 6.0647m ) is dominated almost entirely by the accuracy term and ranks among the worst of all eight methods. This is not a contradiction but a direct manifestation of Equation (14): the raw subspace fix returns the fastest solution precisely because it performs no super-resolution refinement, but the large angular bias it leaves behind, propagated through the accuracy term, dwarfs whatever is saved on the real-time term. The two contributions therefore act as genuinely separate levers—minimizing latency protects only the oscillation term and leaves the accuracy term unbounded. This is the deeper reason why merely minimizing computational latency, as is common in the real-time DOA literature, is insufficient for cooperative localization, and why an estimator must be simultaneously accurate and fast: LOD-PSO is the only compared method that satisfies both requirements, trading a negligible latency increment over FBSS-ESPRIT ( 0.0232s versus 0.0053s , i.e., an oscillation-error increase of only about 0.16m ) for a more-than-tenfold reduction in accuracy error ( 0.5345m versus 6.0645m ).

Situated against prior multi-UAV DOA-assisted localization frameworks, which uniformly assume stationary platforms during the direction-finding-to-report interval, the present contribution is best understood as making the previously hidden real-time term of Equation (14) both explicit and controllable. The displacement-aware reception model of Equations (19)–(23) is what allows the platform-drift contribution to be absorbed at the level of the observation model rather than propagated into the world-coordinate solution, and the isolated column $\Delta \tilde{\epsilon }$ in Table 5 quantifies, for the first time in this setting, the magnitude of the error that the stationary-UAV assumption silently incurs—on the order of 0.05 to 5m depending on the estimator and SNR, since this term scales directly with the algorithmic latency of each method. From a methodological standpoint, the coarse-to-fine architecture of the LOD is likewise a departure from the conventional uniform-overcomplete-dictionary paradigm: by confining the sparse recovery to an SNR-adaptive neighborhood of the FBSS-ESPRIT initialization, the multi-source search space contracts by a factor exceeding $10^{2K}$ , which is the structural mechanism that makes maximum-likelihood-grade super-resolution feasible on embedded ARM-class processors at the sub- 10Hz rate required by field equipment, without GPU acceleration. Equally important, the PSO reformulation converts the sequential, residual-coupled atom selection of OMP-type recovery into a joint evaluation of the complete *K*-source angle vector, which is the key to breaking the source-correlation cascade that degrades greedy methods under coherent signals—a condition that is the norm, not the exception, in farmland multipath environments.

Beyond the specific algorithm, the results carry a broader implication for the deployment of SAGIN-based positioning in precision agriculture. The error budget of Equation (14), together with the geometric feasibility constraints (C1)–(C3), provides a principled design tool that decouples the two levers available to a system integrator: the accuracy term can be reduced by improving the estimator (raising $\eta_{k}$ ), whereas the real-time term can be reduced either by shortening Δt or, geometrically, by maintaining a well-conditioned UAV configuration that bounds ${\left(A^{T}A\right)}^{-1}$ . Because Δt enters quadratically while the geometric amplification enters through the condition number, the analysis suggests that, in high-jitter conditions, investing in either faster onboard computation or tighter formation control yields a larger marginal return than further refining angular accuracy—a quantitative guideline that the raw RMSE-versus-SNR curves alone cannot provide.

From the viewpoint of practical feasibility, the above real-time and accuracy results must also be considered together with UAV autonomy, endurance, and deployment cost. In typical farmland scenarios, the assisting UAVs are not required to perform high-speed trajectory tracking; rather, they mainly need to maintain stable hovering or slow cooperative flight while providing DOA measurements for the UGV. Therefore, the proposed framework is compatible with commercial multi-rotor UAV platforms equipped with lightweight communication, antenna-array, and embedded-computing modules. For short- to medium-duration operations, the available flight time of such UAVs can support continuous localization assistance over a single operation window. For longer smart-tillage tasks, continuous service can be maintained by UAV rotation, scheduled battery replacement, or field-side charging, so that one subset of UAVs performs localization while another subset is recharged or replaced. This deployment strategy prevents UAV endurance from becoming a fundamental limitation of the proposed localization architecture.

The overall system cost is also quantifiable at the level of the main hardware components. A practical implementation mainly consists of multiple commercial UAV platforms, lightweight antenna or receiving arrays, communication modules, and an embedded processing unit. Since the proposed LOD-PSO algorithm achieves the required update rate without GPU acceleration in the simulated setting, the processing unit can potentially be implemented on an ARM-class embedded platform rather than a high-power workstation, which may reduce both cost and energy consumption. In a typical small-scale deployment using three to four assisting UAVs, the dominant cost would come from the UAV platforms and their onboard sensing/communication modules, while the additional algorithmic cost would be limited to the embedded processor and software implementation. Consequently, the proposed system would be more expensive than a single GNSS-only receiver, but it may provide a complementary positioning mechanism for GNSS-degraded or temporarily unavailable conditions. This cost–benefit trade-off may be more acceptable for high-value smart-tillage operations where localization performance can influence operation quality and continuity.

## 7. Conclusions

In the context of climate-smart agriculture (CSA), and in response to the various problems facing unmanned ground vehicles (UGVs) in the field of assisted positioning, this paper proposes a series of innovations to improve both the structure of the positioning model and the assisted-positioning algorithm. In terms of positioning architecture construction, a direction-of-arrival (DOA) estimation-based multi-UAV assisted-positioning architecture is proposed for large-scale smart-farming cultivation scenarios. In terms of the assisted-positioning algorithm, a displacement-aware array signal reception model that integrates the spatio-temporal information of UAVs is constructed. Furthermore, to address the deficiencies of compressed-sensing (CS)-based DOA-assisted positioning algorithms, an adaptive local overcomplete dictionary (LOD) model and a PSO-optimized CS algorithm (LOD-PSO) are proposed.

In Monte Carlo simulations under idealized ULA and narrowband channel models, the proposed method achieves lower DOA RMSE, lower simulated localization error, and lower measured per-frame latency than the state-of-the-art algorithms compared, with its advantages being most pronounced under low-SNR and coherent-source conditions. In other words, the proposed algorithm effectively reduces the consumption of computational resources while maintaining high positioning accuracy in simulation, which in principle makes it well suited to the real-time assisted-positioning requirements of UGVs in large-scale farmland environments. In particular, under conditions of low SNR, a limited number of snapshots, and complex array observations, the proposed method still exhibits good robustness and stability within the simulated settings, supporting the effectiveness of the proposed model and algorithm.

These results suggest the method’s potential for GNSS-outage compensation over large-scale farmland. However, since no field or hardware validation has yet been conducted in this work, any claims regarding operational feasibility, practical robustness, and real-time applicability should be regarded as preliminary and limited to the simulated conditions considered in this study. Hardware-in-the-loop testing and field validation are therefore necessary next steps toward practical deployment. In summary, this study investigates a simulation-based technical approach for accuracy-enhanced and computationally efficient assisted positioning of UGVs in CSA scenarios, while a full field demonstration remains an important direction for future work. 

## Figures and Tables

**Table 1:** Model computational complexity performance analysis.

Algorithm Models	Covariance/Subspace Construction	Parameter Search/Iteration	Overall Computational Complexity
ss-MUSIC [23]	$O(M^{2}Q+M^{3})$	O(LM(M−K))	$O\left(M^{2}Q+M^{3}\right)+O\left(L\;M\left(M-K\right)\right)$
MSCM-OMP [41]	$O(M^{2}Q)$	$O(K\;L_{c}\;M^{2})$	$O\left(M^{2}Q\right)+O\left(K\;L_{c}\;M^{2}\right)$
FOMP [39]	O(MQ)	$O(K\;L_{c}\;M\;Q)$	$O(K\;L_{c}\;M\;Q)$
FBSS-ESPRIT [25]	$O(M^{2}Q+M^{3})$	$O(MK^{2}+K^{3})$	$O(M^{2}Q+M^{3})$
MESA [30]	$O(M^{2}Q)$	$O(T_{m}\;K\;M^{3})$	$O\left(M^{2}Q\right)+O\left(T_{m}\;K\;M^{3}\right)$
MUC-WSF [12]	$O(M^{2}Q+M^{3})$	$O(T_{w}\left(M^{3}+M^{2}K\right))$	$O\left(M^{2}Q+M^{3}\right)+O\left(T_{w}\left(M^{3}+M^{2}K\right)\right)$
MARS [42]	$O(M^{2}Q)$	$O(K\;L_{r}\;M^{2})$	$O\left(M^{2}Q\right)+O\left(K\;L_{r}\;M^{2}\right)$
LOD-PSO	$O(M^{2}Q+M^{3})$	$O(\tilde{P}T_{p}\left(M^{2}K+MK^{2}+K^{3}\right))$	$O\left(M^{2}Q+M^{3}\right)+O\left(\tilde{P}T_{p}\left(M^{2}K+MK^{2}+K^{3}\right)\right)$

**Table 2:** Experimental conditions and parameters.

Conditions	Parameters
The number of array elements	*M*
The number of sources	*K*
The number of snapshots	*Q*
The size of the uniform angular grid (ss-MUSIC)	*L*
The size of the sparse dictionary (MSCM-OMP, FOMP)	$L_{c}$
The size of the reduced search space (MARS)	$L_{r}$
The ADMM iteration number (MESA)	$T_{m}$
The nonlinear-search iteration number (MUC-WSF)	$T_{w}$
**The swarm size (proposed)**	** $\tilde{P}$ **
**The maximum PSO iteration number (proposed)**	** $T_{p}$ **

**Table 3:** Ablation study under non-coherent sources at SNR=2dB .

Config.	Displ. Model	LOD	PSO Recon.	DOA RMSE (°)	Latency (s)
B0: UOD + OMP (baseline)	×	×	×	1.235	0.321
B1: + displ. model	✔	×	×	0.843	0.303
B2: + LOD (still OMP)	✔	✔	×	0.231	0.031
B3: + PSO (full LOD-PSO)	✔	✔	✔	0.100	0.023

**Table 4:** Ablation study under coherent sources at SNR=−5dB .

Config.	Displ. Model	LOD	PSO Recon.	DOA RMSE (°)	Latency (s)
C0: UOD + OMP (baseline)	×	×	×	6.742	0.326
C1: + displ. model	✔	×	×	5.618	0.309
C2: + LOD (still OMP)	✔	✔	×	2.387	0.059
C3: + PSO (full LOD-PSO)	✔	✔	✔	1.450	0.041

**Table 5:** Positioning errors produced by different methods.

Method	SNR (dB)	$\Delta \tilde{\varepsilon }$ (Meter)	t (s)	$\Delta \tilde{\epsilon }$ (Meter)	$\Delta \tilde{\sigma }$ (Meter)
FBSS-ESPRIT [25]	2	6.0645	0.0053	0.0477	6.0647
ss-MUSIC [23]	2	4.8752	0.5910	5.3166	7.2134
FOMP [39]	2	3.9870	0.2480	2.2310	4.5688
MUC-WSF [12]	2	1.5228	0.4210	3.7873	4.0820
MESA [30]	2	1.0522	0.3560	3.2026	3.3710
MARS [42]	2	0.9431	0.0620	0.5578	1.0957
MSCM-OMP [41]	2	0.7634	0.1080	0.9716	1.2356
**LOD-PSO**	**2**	**0.5345**	**0.0232**	**0.2087**	**0.5738**
FBSS-ESPRIT [25]	10	1.9786	0.0049	0.0441	1.9791
ss-MUSIC [23]	10	1.5321	0.5780	5.1997	5.4207
FOMP [39]	10	1.3976	0.2430	2.1860	2.5946
MUC-WSF [12]	10	0.6532	0.4080	3.6704	3.7281
MESA [30]	10	0.5321	0.3470	3.1216	3.1666
MARS [42]	10	0.4715	0.0590	0.5308	0.7100
MSCM-OMP [41]	10	0.4151	0.1140	1.0255	1.1063
**LOD-PSO**	**10**	**0.3223**	**0.0105**	**0.0945**	**0.3359**

## Data Availability

The datasets generated and analyzed during the current study are available from the corresponding authors upon reasonable request, as they will be used in subsequent related studies.

## Appendix A. Detailed Derivation of the Mean-Square Localization Error

In the main text, Equations (11) and (13) give, respectively, the mean-square localization errors induced by the angle-measurement error and by the platform jitter, while the Jacobian structure and the covariance-weighting relations contained therein are stated only in outline. This appendix takes the first-order perturbation of the line-of-sight (LOS) constraint function as a unified starting point: it first recovers the single-LOS error equation and the least-squares localization offset, and then, through second-moment operations, rigorously derives Equations (11) and (13), explaining term by term the physical meaning of the symbols involved. All notation in this appendix is identical to that of the main text.

### Appendix A.1. Preliminaries: LOS Direction and Normal Vectors

Following the definition in Equation (4) of the main text, the LOS direction unit vector and the LOS normal unit vector of the *k*-th UAV are

(A1)
$$u_{k}=\left[\begin{array}{c} \cos\varphi_{k}\\ \sin\varphi_{k} \end{array}\right],\;\;n_{k}=\left[\begin{array}{c} \sin\varphi_{k}\\ -\cos\varphi_{k} \end{array}\right],\;$$

where $u_{k}$ is the unit vector pointing from the UAV toward the target, $n_{k}$ is the unit vector normal to that LOS, and $\varphi_{k}$ is the azimuth measured by the *k*-th UAV. The two vectors satisfy the orthonormality relations

(A2)
$$u_{k}^{T}u_{k}=1,\;\;n_{k}^{T}n_{k}=1,\;\;n_{k}^{T}u_{k}=0,\;$$

in which the first two equations state that both vectors are of unit length and the third states that they are mutually orthogonal. Moreover, the derivative of the normal vector with respect to the azimuth is exactly the LOS direction vector, and the geometric fact that the target lies along the LOS at range $R_{k}$ gives the position decomposition

(A3)
$$\frac{dn_{k}}{d\varphi_{k}}=u_{k},\;\;p-U_{Dk}=R_{k}\;u_{k},\;$$

where $p={\left({\bar{x}}_{t},\;{\bar{y}}_{t}\right)}^{T}$ is the true target position, $U_{Dk}$ is the position of the *k*-th UAV, and $R_{k}=∥p-U_{Dk}∥$ is the range between them. Equations (A2) and (A3) are the fundamental tools for the total-differential operations that follow.

### Appendix A.2. Single-LOS Error Equation and Localization Offset

Following Equation (5) of the main text, define the constraint function of the *k*-th LOS as $f_{k}\left(p\right)=n_{k}^{T}\left(p-U_{Dk}\right)$ , which is identically zero in the ideal error-free case, $f_{k}=0$ . Letting only the azimuth be perturbed while the UAV position retains its nominal value, taking the first-order total differential and enforcing that the constraint remains identically zero yields

(A4)
$${\left(\frac{\partial f_{k}}{\partial p}\right)}^{\;T}dp+\frac{\partial f_{k}}{\partial \varphi_{k}}\;d\varphi_{k}=0,\;$$

where dp is an infinitesimal perturbation of the target position and $d\varphi_{k}$ is an infinitesimal perturbation of the azimuth. Here, the partial derivative with respect to position is directly $\partial f_{k}/\partial p=n_{k}$ , whereas the partial derivative with respect to the azimuth is obtained by means of Equation (A3):

(A5)
$$\frac{\partial f_{k}}{\partial \varphi_{k}}={\left(\frac{dn_{k}}{d\varphi_{k}}\right)}^{\;T}\left(p-U_{Dk}\right)=u_{k}^{T}\left(R_{k}u_{k}\right)=R_{k},\;$$

where the last step uses $u_{k}^{T}u_{k}=1$ ; the result $R_{k}$ indicates that a unit-radian change in azimuth produces a change $R_{k}$ in the constraint quantity, i.e., the farther the range, the stronger the leverage of the angle error on the localization. Substituting both partial derivatives back into Equation (A4), and denoting the physical perturbations $dp=\vec{\Delta \varepsilon }$ and $d\varphi_{k}=\Delta \varphi_{k}$ , recovers Equation (9) of the main text,

(A6)
$$n_{k}^{T}\;\vec{\Delta \varepsilon }+R_{k}\;\Delta \varphi_{k}=0,\;$$

where $\vec{\Delta \varepsilon }$ is the two-dimensional localization offset induced by the angle-measurement error and $\Delta \varphi_{k}={\hat{\varphi }}_{k}-\varphi_{k}$ is the angle-measurement error of the *k*-th UAV. Stacking Equation (A6) over k=1, 2, 3 into the matrix form $A\;\vec{\Delta \varepsilon }=-D\;\Delta \varphi$ , where $A={\left[n_{1},\;n_{2},\;n_{3}\right]}^{T}$ is the observation matrix of Equation (6), $D=\mathrm{diag}(R_{1},\;R_{2},\;R_{3})$ is the diagonal range matrix, and $\Delta \varphi ={\left[\Delta \varphi_{1},\;\Delta \varphi_{2},\;\Delta \varphi_{3}\right]}^{T}$ is the angle-error vector. Since the number of equations exceeds the number of unknowns, taking the least-squares solution and setting the gradient of the sum of squared residuals to zero gives the normal equations $A^{T}A\;\vec{\Delta \varepsilon }=-A^{T}D\;\Delta \varphi$ ; left-multiplying both sides by ${\left(A^{T}A\right)}^{-1}$ (whose invertibility is guaranteed by constraint (C2) recovers Equation (10) of the main text,

(A7)
$$\vec{\Delta \varepsilon }=-{\left(A^{T}A\right)}^{-1}A^{T}D\;\Delta \varphi =J_{\varphi }\;\Delta \varphi ,\;$$

where $J_{\varphi }=-{\left(A^{T}A\right)}^{-1}A^{T}D\in R^{2\times 3}$ is the angular Jacobian matrix. This completes the preparation of the linear mapping required to prove Equation (11).

### Appendix A.3. Mean-Square Localization Error Due to Angle-Measurement

By Equation (A7), the localization offset $\vec{\Delta \varepsilon }$ is a linear function of the random angle-measurement error Δφ . Using the identity ${∥x∥}^{2}=\mathrm{tr}\left(xx^{T}\right)$ relating norm and trace, together with the interchangeability of the trace and expectation operators, the mean-square localization error is written as

(A8)
$$E\left[∥\vec{\Delta \varepsilon }{∥}^{2}\right]=E\left[\mathrm{tr}(\vec{\Delta \varepsilon }\;{\vec{\Delta \varepsilon }}^{T})\right]=\mathrm{tr}\left(E[\vec{\Delta \varepsilon }\;{\vec{\Delta \varepsilon }}^{T}]\right),\;$$

where E[·] denotes the expectation over the random angle-measurement error and tr(·) is the matrix trace. Substituting Equation (A7) into the outer product and moving the constant matrix $J_{\varphi }$ , which is independent of the random quantity, outside the expectation gives

(A9)
$$E\left[\vec{\Delta \varepsilon }\;{\vec{\Delta \varepsilon }}^{T}\right]=J_{\varphi }\;E\left[\Delta \varphi \;\Delta \varphi^{T}\right]\;J_{\varphi }^{T}=J_{\varphi }\;C_{\varphi }\;J_{\varphi }^{T},\;$$

where $C_{\varphi }=E\left[\Delta \varphi \;\Delta \varphi^{T}\right]=\mathrm{diag}\left(\sigma_{\varphi_{1}}^{2},\;\sigma_{\varphi_{2}}^{2},\;\sigma_{\varphi_{3}}^{2}\right)$ is the angle-error covariance matrix, whose diagonal structure follows from the assumption that the angle-measurement errors of the individual UAVs are zero-mean and mutually uncorrelated, and $\sigma_{\varphi_{k}}^{2}$ is the angle-measurement variance of the *k*-th UAV. Substituting $J_{\varphi }=-{\left(A^{T}A\right)}^{-1}A^{T}D$ into Equation (A9) and taking the trace, noting that D is a symmetric diagonal matrix ( $D^{T}=D$ ) and that the negative sign cancels upon squaring, yields Equation (11) of the main text,

(11)
$$E\left[∥\vec{\Delta \varepsilon }{∥}^{2}\right]=\mathrm{tr}\left(J_{\varphi }C_{\varphi }J_{\varphi }^{T}\right)=\mathrm{tr}\left[{\left(A^{T}A\right)}^{-1}A^{T}DC_{\varphi }DA{\left(A^{T}A\right)}^{-1}\right].$$

This completes the proof. Its physical structure is clearly discernible: the inner factor $DC_{\varphi }D$ is diagonal, its *k*-th diagonal element being $R_{k}^{2}\sigma_{\varphi_{k}}^{2}$ , which shows that each UAV’s contribution is proportional to the product of its squared range and angle-measurement variance (i.e., the LOS transverse-displacement variance); the outer factor ${\left(A^{T}A\right)}^{-1}$ is governed by the geometric dilution of precision (GDOP) of Equation (8), aggregating the transverse displacements of the UAVs into the final localization error with a weighting reflecting the quality of the configuration. This term depends only on the angle-measurement accuracy and the three-UAV geometry and is independent of time.

### Appendix A.4. Injection of Platform-Jitter Error and the Localization

Consider now the opposite situation, in which the azimuths retain their nominal values while the UAV positions drift owing to hovering jitter. Within the total latency Δt , the position of the *k*-th UAV drifts from $U_{Dk}$ to $U_{Tk}$ , with displacement $\delta_{k}=U_{Tk}-U_{Dk}$ . Taking the partial derivative of the constraint function with respect to the UAV position gives $\partial f_{k}/\partial U_{Dk}=-n_{k}$ (the negative sign arising because $U_{Dk}$ enters the constraint as $-U_{Dk}$ ), and the first-order total differential that keeps the constraint satisfied gives

(A10)
$$n_{k}^{T}\;\vec{\Delta \epsilon }+{\left(-n_{k}\right)}^{T}\delta_{k}=0\;⟹\;n_{k}^{T}\;\vec{\Delta \epsilon }=n_{k}^{T}\delta_{k}≜w_{\mathrm{osc},\;k},\;$$

where $\vec{\Delta \epsilon }$ is the two-dimensional localization offset induced by jitter and $w_{\mathrm{osc},\;k}=n_{k}^{T}\delta_{k}$ is the scalar projection of the position drift onto the LOS normal. Since $n_{k}⊥u_{k}$ (Equation (A2)), the radial drift along the LOS is filtered out by the inner product, and only the component normal to the LOS injects error. Stacking Equation (A10) over the three UAVs into $A\;\vec{\Delta \epsilon }=w_{\mathrm{osc}}$ and taking the least-squares solution recovers Equation (12) of the main text,

(A11)
$$\vec{\Delta \epsilon }={\left(A^{T}A\right)}^{-1}A^{T}w_{\mathrm{osc}},\;\;w_{\mathrm{osc}}={\left[w_{\mathrm{osc},\;1},\;w_{\mathrm{osc},\;2},\;w_{\mathrm{osc},\;3}\right]}^{T},\;$$

where $w_{\mathrm{osc}}\in R^{3}$ is the vector formed by the normal jitter projections of the UAVs, the remaining symbols being as defined above. This completes the preparation of the mapping required to prove Equation (13).

### Appendix A.5. Mean-Square Localization Error Due to Platform Jitter

Taking the mean-square expectation of Equation (A11) in the same manner as in Appendix A.3 gives

(A12)
$$E\left[∥\vec{\Delta \epsilon }{∥}^{2}\right]=\mathrm{tr}\left[{\left(A^{T}A\right)}^{-1}A^{T}\;E\left[w_{\mathrm{osc}}w_{\mathrm{osc}}^{T}\right]\;A{\left(A^{T}A\right)}^{-1}\right],\;$$

where $E[w_{\mathrm{osc}}w_{\mathrm{osc}}^{T}]$ is the covariance matrix of the normal jitter projections. Assuming that the jitter of each UAV is isotropic and mutually independent, and that the drift is accumulated from a zero-mean jitter velocity over the latency Δt , the second moment of the displacement is

(A13)
$$E\left[\delta_{i}\delta_{j}^{T}\right]=\sigma_{\mathrm{osc},\;k}^{2}\;\Delta t^{2}\;I_{2}\;\delta_{ij},\;$$

where $\sigma_{\mathrm{osc},\;k}^{2}$ is the variance of the jitter velocity of the *k*-th UAV, Δt is the total latency from direction finding to reporting, $I_{2}$ is the 2×2 identity matrix, and $\delta_{ij}$ is the Kronecker delta (zero for i≠j owing to inter-UAV independence). Consequently, the (k, k) element of the covariance matrix $E[w_{\mathrm{osc}}w_{\mathrm{osc}}^{T}]$ is

(A14)
$$E\left[w_{\mathrm{osc},\;k}^{2}\right]=n_{k}^{T}\;E\left[\delta_{k}\delta_{k}^{T}\right]\;n_{k}=\sigma_{\mathrm{osc},\;k}^{2}\Delta t^{2}\;n_{k}^{T}n_{k}=\sigma_{\mathrm{osc},\;k}^{2}\Delta t^{2},\;$$

where the last step uses $n_{k}^{T}n_{k}=1$ (Equation (A2)); the off-diagonal elements vanish owing to inter-UAV independence. Hence $E\left[w_{\mathrm{osc}}w_{\mathrm{osc}}^{T}\right]=\mathrm{diag}\left(\sigma_{\mathrm{osc},\;k}^{2}\right)\;\Delta t^{2}$ . Substituting back into Equation (A12) yields Equation (13) of the main text,

(13)
$$E\left[∥\vec{\Delta \epsilon }{∥}^{2}\right]=\mathrm{tr}\left[{\left(A^{T}A\right)}^{-1}A^{T}\mathrm{diag}\left(\sigma_{\mathrm{osc},\;k}^{2}\right)A{\left(A^{T}A\right)}^{-1}\right]\;\Delta t^{2}.$$

This completes the proof. The expression explicitly shows that the mean-square jitter error is proportional to the squared latency $\Delta t^{2}$ ; that is, any processing or communication delay is amplified by the platform jitter into an irreducible localization error.

Furthermore, Equation (13) can be unified with the angular-Jacobian framework of Equation (11). From Equation (A10), the normal position drift of a UAV is equivalent to an angular perturbation of magnitude $\Delta \varphi_{k}^{\mathrm{osc}}=-w_{\mathrm{osc},\;k}/R_{k}$ with variance $\sigma_{\mathrm{osc},\;k}^{2}\Delta t^{2}/R_{k}^{2}$ . Treating this as an equivalent angle-error covariance $C_{\varphi }^{\mathrm{osc}}=\mathrm{diag}\left(\sigma_{\mathrm{osc},\;k}^{2}\Delta t^{2}/R_{k}^{2}\right)$ and substituting it into the Jacobian structure of Equation (11), the diagonal element of the inner factor $DC_{\varphi }^{\mathrm{osc}}D$ is exactly $R_{k}^{2}\cdot \sigma_{\mathrm{osc},\;k}^{2}\Delta t^{2}/R_{k}^{2}=\sigma_{\mathrm{osc},\;k}^{2}\Delta t^{2}$ , verifying that the result is fully equivalent to Equation (13). This equivalent viewpoint brings the two error sources under the same Jacobian $J_{\varphi }$ and constitutes precisely the mathematical basis for the additive decomposition of Equation (14) into an “accuracy term” and a “real-time term”: the jitter contribution scales in the equivalent angle-error budget as $\sum_{k}\sigma_{\mathrm{osc},\;k}^{2}\Delta t^{2}/R_{k}^{2}$ , clearly exhibiting its dual character of growing with the squared latency and decaying inversely with the squared range.

## References

[1] Usman B.Y., Gaya A.D., Kanya R.A., Abdullahi R., Binta M. Advancing Climate-Smart Agriculture in Nigeria: Using Hyperspectral and Remote Sensing Technologies to Bridge Mechanisation and Smallholder Farming in Jigawa State. Proceedings of the 2025 IEEE International Humanitarian Technology Conference (IHTC).

[2] Kuchanskyi O., Neftissov A., Biloshchytskyi A., Andrashko Y., Vatskel V. Development of Smart Irrigation System Based on Climate-Smart Agricultural Practices. Proceedings of the 2025 IEEE Conference on Technologies for Sustainability (SusTech).

[3] Song B., Giamou M., Yan P., Xia C., Hsu L.-T. (2026). Certifiable Alignment of GNSS and Local Frames via Lagrangian Duality. IEEE Robot. Autom. Lett..

[4] Wang Q., Shan S., Zhang H., Yao Z. (2026). Context-Aware Adaptive Filtering for GNSS/INS Integration via Multiscale Time–Frequency Feature Fusion. IEEE Trans. Instrum. Meas..

[5] Ji H.-J., Zhang J.P., Guo L.X., Wei Y.W., Leng J.Q., Zhang Y., Li Q.L., Guo X.M., Zhang Y.S. (2026). DenseRefrception: A Densely Connected Perception Framework for Passive Retrieval of Ocean Refractive Environments via GPS Scattered Signals. IEEE Trans. Geosci. Remote Sens..

[6] He Y., Liao G., He Z., Chen Z.N. (2025). A 3D-FSS-Based and Front-Feeding Shared-Aperture Base Station Antenna for 5G Mobile Communications. IEEE Trans. Antennas Propag..

[7] Mai R., Nguyen D.H.N., Le-Ngoc T. (2015). Linear Precoding Game for MIMO MAC with Dynamic Access Point Selection. IEEE Wirel. Commun. Lett..

[8] Ge H., Meng G., Li B. (2024). Zero-Reconvergence PPP for Real-Time Low-Earth Satellite Orbit Determination in Case of Data Interruption. IEEE J. Sel. Top. Appl. Earth Obs. Remote Sens..

[9] Wang H., Wang X., Lan X., Su T., Wan L. (2023). BSBL-Based Auxiliary Vehicle Position Analysis in Smart City Using Distributed MEC and UAV-Deployed IoT. IEEE Internet Things J..

[10] Miao W., Luo C., Min G., Mi Y., Yu Z. (2021). Location-Based Robust Beamforming Design for Cellular-Enabled UAV Communications. IEEE Internet Things J..

[11] Cong J., Wang X., Yan C., Yang L.T., Dong M., Ota K. (2023). CRB Weighted Source Localization Method Based on Deep Neural Networks in Multi-UAV Network. IEEE Internet Things J..

[12] Wang X., Yang L.T., Meng D., Dong M., Ota K., Wang H. (2022). Multi-UAV Cooperative Localization for Marine Targets Based on Weighted Subspace Fitting in SAGIN Environment. IEEE Internet Things J..

[13] Li W., Ma Y., Liu J., Xi L., Ma X., Liu X. 3D Reconstruction Strategy of Vehicle Targets Based on Multi-Angle Observation Using UAV Swarm SAR. Proceedings of the 2025 10th International Conference on Intelligent Computing and Signal Processing (ICSP).

[14] Qian M., Chen W., Sun R. (2024). A Maneuvering Target Tracking Algorithm Based on Cooperative Localization of Multi-UAVs with Bearing-Only Measurements. IEEE Trans. Instrum. Meas..

[15] Dong W., Ju Z., Jiao H., Liu Z., Chen R., Chen L. (2026). Beam-Switching-Based Joint DOA and TOA Acquisition and Tracking Using Commercial 5G NR Signals for Outdoor Positioning. IEEE Trans. Wirel. Commun..

[16] Ling L., Tang M., Wang J., Tan W., Li C. (2026). Error-Based Filtering and Adaptive Weighting for Real-Time DL-TDoA Localization. IEEE Trans. Instrum. Meas..

[17] He L., Fang Z., He Y., Meng F., Shi G. (2026). DFRP: Multipath Robust Dual-Feature RFID Positioning Using Phase and RSSI for Food-Industry Warehousing. IEEE Trans. Instrum. Meas..

[18] Zhou C., Liu J., Sheng M., Zheng Y., Li J. (2021). Exploiting Fingerprint Correlation for Fingerprint-Based Indoor Localization: A Deep Learning Based Approach. IEEE Trans. Veh. Technol..

[19] Krim H., Viberg M. (1996). Two decades of array signal processing research: The parametric approach. IEEE Signal Process. Mag..

[20] Capon J. (1969). High-resolution frequency-wavenumber spectrum analysis. Proc. IEEE.

[21] Schmidt, Otto R. (1982). A sIgnal Subspace Approach to Multiple Emitter Location and Spectral Estimation. Ph.D. Thesis.

[22] Yan F., Jin M., Zhou H., Wang J., Liu S. (2020). A Low-degree root-MUSIC algorithm for fast DOA estimation based on variable substitution technique. Sci. China. Inf. Sci..

[23] Liu C.-L., Vaidyanathan P.P. (2015). Remarks on the Spatial Smoothing Step in Coarray MUSIC. IEEE Signal Process. Lett..

[24] Roy R., Kailath T. (1989). ESPRIT-estimation of signal parameters via rotational invariance techniques. IEEE Trans. Acoust. Speech Signal Process..

[25] Yang Z. (2023). Nonasymptotic Performance Analysis of ESPRIT and Spatial-Smoothing ESPRIT. IEEE Trans. Inf. Theory.

[26] Chen H., Suzuki M. On non-uniqueness of stochastic ML estimation of DOA and its best solution. Proceedings of the 2007 International Symposium on Intelligent Signal Processing and Communication Systems.

[27] Chen H., Suzuki M. (2010). Exact formulation for stochastic ML estimation of DOA. IEICE Trans. Fundam. Electron. Commun. Comput. Sci..

[28] Palanisamy P., Kalyanasundaram N., Raghunandan A. (2009). A new DOA estimation algorithm for wideband signals in the presence of unknown spatially correlated noise. Signal Process..

[29] Simeoni M., Hurley P. SIML: Sieved Maximum Likelihood for Array Signal Processing. Proceedings of the ICASSP 2021–2021 IEEE International Conference on Acoustics, Speech and Signal Processing (ICASSP).

[30] Yang Z., Chen X., Wu X. (2023). A Robust and Statistically Efficient Maximum-Likelihood Method for DOA Estimation Using Sparse Linear Arrays. IEEE Trans. Aerosp. Electron. Syst..

[31] Zamani H., Zayyani H., Marvasti F. (2016). An Iterative Dictionary Learning-Based Algorithm for DOA Estimation. IEEE Commun. Lett..

[32] Fu H., Dai F., Hong L. (2023). Two-Dimensional Off-Grid DOA Estimation with Metasurface Aperture Based on MMV Sparse Bayesian Learning. IEEE Trans. Instrum. Meas..

[33] Wagner M., Park Y., Gerstoft P., Tang W.-G., Jiang H., Pang S.-X. (2019). Grid-Free DOD and DOA Estimation for MIMO Radar via Duality-Based 2D Atomic Norm Minimization. IEEE Access.

[34] Dong N., Zhang L., Zhou H., Li X., Wu S., Liu X. (2023). Two-Stage Fast Matching Pursuit Algorithm for Multi-Target Localization. IEEE Access.

[35] Shi W., He Q., Wu H. (2023). Generalized Spatial-Temporal Coprime Sampling for Joint DOA and Doppler Estimation. IEEE Geosci. Remote Sens. Lett..

[36] Candes E.J., Romberg J., Tao T. (2006). Robust uncertainty principles: Exact signal reconstruction from highly incomplete frequency information. IEEE Trans. Inf. Theory.

[37] Candes E.J., Tao T. (2005). Decoding by linear programming. IEEE Trans. Inf. Theory.

[38] Liu L., Du X., Cheng L. (2013). Stable Signal Recovery via Randomly Enhanced Adaptive Subspace Pursuit Method. IEEE Signal Process. Lett..

[39] Dehghani M., Aghababaiyan K. (2018). FOMP algorithm for direction of arrival estimation. Phys. Commun..

[40] Liu X., Wang H., Shen X., Dong H., Jing H. Joint Sparse with Generalized Orthogonal Matching Pursuit for Off-Grid Wideband DOA Estimation. Proceedings of the 2019 IEEE International Conference on Signal Processing, Communications and Computing (ICSPCC).

[41] Wu Y., Li C., Hou Y.T., Lou W. (2024). A Real-Time Super-Resolution DoA Estimation Algorithm for Automotive Radar Sensor. IEEE Sens. J..

[42] Hoang T.-D., Huang X., Qin P. (2025). Low-Complexity Direction-of-Arrival Estimation with Orthogonal Matching Pursuit for Large-Scale Lens Antenna Array. IEEE Trans. Commun..

[43] Xu Z., Chen Y., Zhang P. (2023). A Sparse Uniform Linear Array DOA Estimation Algorithm for FMCW Radar. IEEE Signal Process. Lett..

[44] Ye Z., Xu X. (2007). DOA Estimation by Exploiting the Symmetric Configuration of Uniform Linear Array. IEEE Trans. Antennas Propag..

[45] Zhang T., Yuan M., Wang L., Tang H., Niu X. (2024). A Robust and Efficient IMU Array/GNSS Data Fusion Algorithm. IEEE Sens. J..

[46] Tran L.C., Le A.T., Huang X., Dutkiewicz E., Ngo D., Taparugssanagorn A. (2023). Complexity Reduction for Hybrid TOA/AOA Localization in UAV-Assisted WSNs. IEEE Sens. Lett..

