# Correction: Zhang et al. A Robust CS-DOA Method for Multi-UAV-Assisted Agricultural Vehicle Localization in Smart Tillage. *Sensors* 2026, *26*, 5377

**DOI:** 10.3390/s26185906

**Published:** 2026-09-18

**Authors:** Jingyao Zhang, Ningning Ma, Heyang Li, Yancong Wang, Xiaobo Zhang, Zaiwang Lu, Lei Li, Haihua Chen, Yucheng Zhang

**Affiliations:** 1 Institute of Computing Technology, Chinese Academy of Sciences, Beijing 100190, China; zhangjingyao23p@ict.ac.cn (J.Z.); liheyang23p@ict.ac.cn (H.L.); wangyancong@ict.ac.cn (Y.W.); zhangxiaobo24b@ict.ac.cn (X.Z.); luzaiwang21b@ict.ac.cn (Z.L.); lilei22p@ict.ac.cn (L.L.); 2 University of Chinese Academy of Sciences, Beijing 100049, China; 3 Institute of Applied Ecology, Chinese Academy of Sciences, Shenyang 110164, China; ma_ningning23@163.com

## Removal of an Author

Feng Dai was removed from the list of authors in the original publication [1]. The corrected Author Contributions statement appears here. The authors state that the scientific conclusions are unaffected. This correction was approved by the Academic Editor. The original publication has also been updated.

**Author Contributions:** Conceptualization, J.Z. and H.C.; system architecture and error-propagation theory (Section 2 and Appendix A), J.Z. and Y.Z.; displacement-aware receive model (Section 3), J.Z. and H.L.; LOD construction and Monte Carlo phase-transition study (Section 4.1), N.M. and X.Z.; PSO reconstruction algorithm and its implementation (Section 4.2), J.Z. and Z.L.; simulation software and reproducibility, N.M. and L.L.; complexity analysis (Section 5.2), J.Z.; position-error experiments (Section 5.4), Y.W.; agronomic requirements and field-scenario framing (Section 2), N.M.; supervision and funding acquisition, H.C. and Y.Z.; drafting of the manuscript, J.Z.; review and editing, all authors. All authors have read and agreed to the published version of the manuscript.
